# ORP4L is essential for T-cell acute lymphoblastic leukemia cell survival

**DOI:** 10.1038/ncomms12702

**Published:** 2016-09-01

**Authors:** Wenbin Zhong, Qing Yi, Bing Xu, Shiqian Li, Tong Wang, Fupei Liu, Biying Zhu, Peter R. Hoffmann, Guangju Ji, Pingsheng Lei, Guoping Li, Jiwei Li, Jian Li, Vesa M. Olkkonen, Daoguang Yan

**Affiliations:** 1 Key Laboratory of Functional Protein Research of Guangdong Higher Education Institutes, Department of Biotechnology, College of Life Science and Technology, Jinan University, Guangzhou 510632, China; 2 Department of Cancer Biology, Lerner Research Institute, Cleveland Clinic, Cleveland, Ohio 44195, USA; 3 Department of Hematology, Nanfang Hospital, Southern Medical University, Guangzhou 510515, China; 4 Department of Cell and Molecular Biology, John A. Burns School of Medicine, University of Hawaii, Honolulu, Hawaii 96813, USA; 5 National Laboratory of Biomacromolecules, Institute of Biophysics, Chinese Academy of Sciences, Beijing 100101, China; 6 State Key Laboratory of Bioactive Substances and Functions of Natural Medicines, Chinese Academy of Medical Sciences, Beijing 100050, China; 7 The Key Laboratory of Geriatrics, Beijing Hospital, National Center of Gerontology & Beijing Institute of Geriatrics, Ministry of Health, Beijing 100730, China; 8 Minerva Foundation Institute for Medical Research, Biomedicum 2U, Helsinki FI-00290, Finland

## Abstract

Metabolic pathways are reprogrammed in cancer to support cell survival. Here, we report that T-cell acute lymphoblastic leukemia (T-ALL) cells are characterized by increased oxidative phosphorylation and robust ATP production. We demonstrate that ORP4L is expressed in T-ALL but not normal T-cells and its abundance is proportional to cellular ATP. ORP4L acts as an adaptor/scaffold assembling CD3ɛ, Gα_q/11_ and PLCβ3 into a complex that activates PLCβ3. PLCβ3 catalyzes IP_3_ production in T-ALL as opposed to PLCγ1 in normal T-cells. Up-regulation of ORP4L thus results in a switch in the enzyme responsible for IP_3_-induced endoplasmic reticulum Ca^2+^ release and oxidative phosphorylation. ORP4L knockdown results in suboptimal bioenergetics, cell death and abrogation of T-ALL engraftment *in vivo*. In summary, we uncovered a signalling pathway operating specifically in T-ALL cells in which ORP4L mediates G protein-coupled ligand-induced PLCβ3 activation, resulting in an increase of mitochondrial respiration for cell survival. Targeting ORP4L might represent a promising approach for T-ALL treatment.

## Abstract (web summary)

Lymphocytic leukaemia cells are characterized by high respiratory rates. Here, the authors report that the oxysterol-binding protein ORPL4 sustains mitochondrial respiration in T-cell acute lymphoblastic leukaemia cells by regulating Ca^2+^ release from the endoplasmic reticulum.

## Graphical abstract

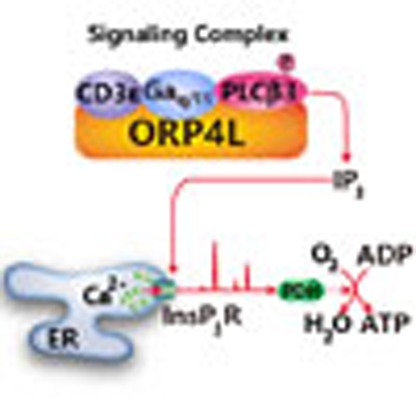

Reprogramming of metabolic pathways by oncogenic signalling has emerged as a hallmark of cancer cells and may offer attractive targets for anticancer strategies^1^,^2^,^3^. T-cell acute lymphoblastic leukemia (T-ALL) is one of the deadliest and most aggressive hematological malignancies. Despite progress in intensive chemotherapy has achieved a 5-year event-free survival rate of 60–70% in children and 30–40% in adults, the success of the treatment is limited with 20–25% of paediatric and over 50% of adult patients with T-ALL exhibiting resistance to therapy and relapse^4^,^5^. Enhanced molecular understanding of T-ALL biology will ultimately facilitate a targeted therapy driven approach that can improve survival of refractory T-ALL patients.

Ca^2+^ released from the endoplasmic reticulum (ER) is taken up by mitochondria through the mitochondrial Ca^2+^ uniporter (MCU)^6^,^7^,^8^. This process provides reducing equivalents to support oxidative phosphorylation^9^,^10^ through the activation of three intramitochondrial dehydrogenases^10^,^11^ and ATP synthase^12^.

Phospholipase Cs (PLCs) are a class of enzymes that execute an initial step in intracellular Ca^2+^ signaling^13^,^14^. In normal T-cells, PLCγ1 is a dominant enzyme controlling IP_3_ production and Ca^2+^ homeostasis^15^. PLCγ1 is activated through the TCR complex independent of G protein transduction. In contrast, anti-CD3-induced Ca^2+^ release was not impaired in T-ALL cells lacking PLCγ1 (ref. 16), suggesting that other components beyond PLCγ1 may be responsible for Ca^2+^ release in these cells. PLCβs are canonical downstream targets of the Gq subfamily of G protein-coupled receptors (GPCRs) and have been reported to regulate Ca^2+^ release upon anti-CD3 stimulation in Jurkat T-cells^16^. This raises the issue of the role that PLCβs versus PLCγ1 play in Ca^2+^ homeostasis of T-ALL cells and how PLCβs are activated upon anti-CD3 stimulation of these cells. These heretofore unresolved fundamental issues are crucial for understanding the signalling pathways required for PLC activation and Ca^2+^ homeostasis in T-ALL cells.

Oxysterol-binding protein (OSBP)-related protein 4 (ORP4; also known as OSBP2) is present as three major variants, ORP4L, ORP4M and ORP4S^17^,^18^. ORP4 is expressed constitutively in brain, heart and testis, but is virtually absent from other human and mouse tissues^17^,^19^. ORP4 knockout mice exhibit teratozoospermia due to death of developing spermatozoa, indicating that ORP4 is essential for the survival of specific cell populations^19^. Early studies reported that ORP4L was detectable in leukocytes from patients with chronic myeloid leukemia but not healthy donors^20^,^21^. Recent reports have consistently indicated that ORP4L is involved in tumour cell proliferation and survival^18^ and a target of the natural anti-proliferative steroidal saponin, OSW-1 (ref. 22), suggesting the involvement of ORP4L in control of oncogenic cell growth.

In the present study, we report the importance of elevated ORP4L expression in G protein-coupled ligand-induced PLC activation in T-ALL cells and identify ORP4L as a major node in Ca^2+^ homeostasis, bioenergetics and survival of these cells.

## Results

### Elevated ORP4L expression is related to increased respiratory rate

**Figure 1:**
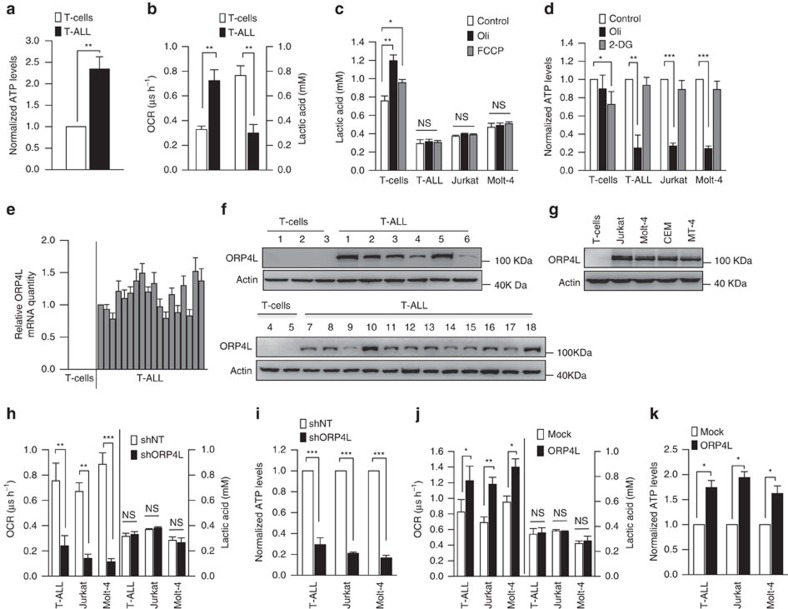
Elevated ORP4L expression is concurrent with aberrant energy metabolism in T-ALL cells. (**a**) ATP levels were measured in normal T-cells (*n*=5) and primary T-ALL cells (*n*=8). (**b**) Baseline OCR and lactic acid levels in normal T-cells (*n*=4) and primary T-ALL cells (*n*=6). (**c**) Lactic acid levels in normal T-cells (*n*=4), primary T-ALL cells (*n*=6), Jurkat T-cells and Molt-4 cells after treatment of oligomycin (Oli, 5 μM) or FCCP (5 μM) for 4 h. (**d**) ATP levels were measured in normal T-cells (*n*=4), primary T-ALL cells (*n*=3), Jurkat T-cells and Molt-4 cells after treatment with oxidative phosphorylation inhibitor oligomycin (Oli, 5 μM) or glycolysis inhibitor (2-DG, 5 μM) for 6 h. (**e**,**f**) ORP4L mRNA (**e**) and protein expression (**f**) in normal T-cells and primary T-ALL cells. (**g**) ORP4L expression in normal T-cells and T-ALL cell lines. (**h**) Baseline OCR and lactic acid levels in primary T-ALL cells (*n*=3), Jurkat T-cells and Molt-4 cells with ORP4L knockdown. (**i**) ATP levels in primary T-ALL cells (*n*=3), Jurkat T-cells and Molt-4 cells with ORP4L knockdown. (**j**) Baseline OCR and lactic acid levels in primary T-ALL cells (*n*=3), Jurkat T-cells and Molt-4 cells with ORP4L overexpression. (**k**) ATP levels in primary T-ALL cells (*n*=3), Jurkat T-cells and Molt-4 cells with ORP4L overexpression. The data represent mean±s.d. value from an experiment performed in triplicate. *P<0.05, **P<0.01, ***P<0.001, NS, not significant, Student's *t* test.

In contrast to most cancer cells that rely on ‘aerobic glycolysis' as the major source of ATP, leukocytes from patients with acute lymphocytic leukemia are characterized by high respiratory rates and low aerobic glycolysis^23^. To gain insights into the metabolic regulation of T-ALL cells, we first performed bioenergetics analyses in normal T-cells and primary T-ALL cells. We found significantly elevated cellular ATP levels (Fig. 1a) and increases of ATP in both the mitochondria and the cytosol (Supplementary Fig. 1a,b) of primary T-ALL cells. This robust energy production is further underlined by the increased rate of basic oxygen consumption (OCR; Fig. 1b) accompanied by a significantly increased reactive oxygen species (ROS) production (Supplementary Fig. 1c). In most other types of cancer, aerobic glycolysis characterized by lactate production is hyperactive^24^. This does not appear to be the case in primary T-ALL cells (Fig. 1b). Oligomycin (Oli) and FCCP inhibit mitochondrial respiration and force cells to the glycolytic pathway to maintain energy supply; treatment with these agents is indicative of the reserve cellular glycolytic potential. Normal T-cells displayed a significant increase of the aerobic glycolysis under oligomycin and FCCP treatment (Fig. 1c). Whereas, primary T-ALL cells and T-ALL cell lines including Jurkat, Molt-4, CEM and MT-4 cells showed a decreased potential to upregulate the glycolytic machinery (Fig. 1c; Supplementary Fig. 1d). Furthermore, the glycolysis inhibitor 2-deoxy-d-glucose (2-DG) slightly decreased ATP generation in normal T-cells (Fig. 1d). In contrast, primary T-ALL cells and T-ALL cell lines exhibited a significant decrease of ATP upon oligomycin treatment, but were resistant to a drop of cellular ATP induced by 2-DG (Fig. 1d; Supplementary Fig. 1e).

Because previous studies suggested expression of ORP4L in leukocytes from patients with chronic myeloid leukemia^20^,^21^, we compared ORP4L expression in normal T-cells and primary T-ALL cells. High levels of ORP4L mRNA and protein were detected in all 18 primary T-ALL specimens (Supplementary Table 1) but not in normal T-cells (Fig. 1e,f). All of the T-ALL cell lines used above also displayed high ORP4L expression (Fig. 1g). Next, we infected primary T-ALL cells and cell lines with lentivirus carrying a small hairpin RNA (shRNA) targeting ORP4L (shORP4L) or ORP4L cDNA, and confirmed the knockdown and overexpression of ORP4L in these cells (Supplementary Fig. 2a–d). Surprisingly, ORP4L depletion in primary T-ALL and cell lines resulted in a reduction of cellular OCR (Fig. 1h; Supplementary Fig. 2e) and ATP levels (Fig. 1i; Supplementary Fig. 2f), whereas ORP4L overexpression increased these parameters (Fig. 1j,k; Supplementary Fig. 2g,h). To exclude off-target effects of ORP4L shRNA, we also performed rescue experiments in ORP4L knockdown Jurkat T-cells, overexpression of ORP4L abolished the OCR and ATP decrease upon ORP4L knockdown (Supplementary Fig. 2i). These results indicated that ORP4L is required for the energy homeostasis of T-ALL cells.

Aberrant Notch-1 signalling has a major role in the pathogenesis of T-ALL, as more than 60% of T-ALL cases harbour activating mutations in the *NOTCH-1* gene^25^. Most T-ALL cell lines harbouring activating mutations in *NOTCH-1* fail to respond to small-molecule γ-secretase inhibitors (GSIs) therapy, owing to mutational loss of the phosphatase and tensin homolog (PTEN) tumour suppressor^26^. We detected Notch-1 and PTEN status in all 18 T-ALL primary samples. Among the 18 cases, 10 have activating mutations that involve the extracellular heterodimerization domain and/or the C-terminal PEST domain of NOTCH-1, and 7 of the 18 samples display PTEN loss (Supplementary Fig. 3a). However, the expression of ORP4L is independent of the Notch-1 and PTEN status. Recently, PTEN-null T-ALL cells were shown to display upregulated glycolysis^27^ as compared with PTEN-positive cells. Jurkat, CEM and Molt-4 are PTEN-null cell lines, and MT-4 cells are PTEN-positive (Supplementary Fig. 3b). However, all of these cell lines were unable to resort to glycolysis in response to uncoupling of respiration (Fig. 1c,d; Supplementary Fig. 1d,e). These results support the notion that T-ALL cells may paradoxically depend more on mitochondrial oxidative phosphorylation than glycolysis to meet their energy demands.

### ORP4L assembles CD3ɛ with Gα_q/11_ and PLCβ3 into a signalling complex

**Figure 2:**
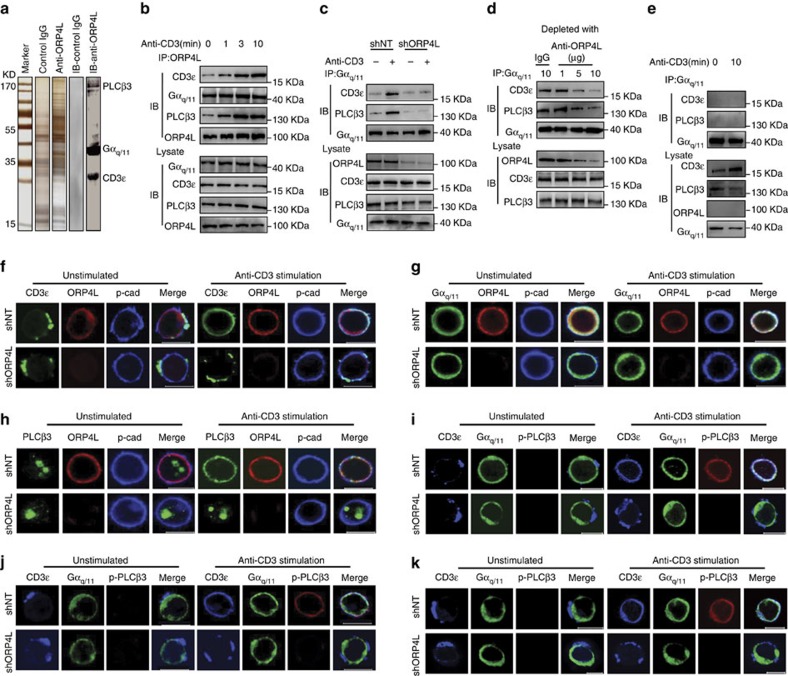
ORP4L facilitates assembly of a signalling complex in T-ALL cells. (**a**) SDS–PAGE gel silver-stained for control IgG and anti-ORP4L immunoprecipitated proteins from Jurkat T-cells. The lane marked IB displays control IgG and anti-ORP4L immunoprecipitated proteins analyzed by western blot with anti-PLCβ3, Gα_q/11_ and CD3ɛ antibodies simultaneously. (**b**) Co-immunoprecipitation analysis of ORP4L binding proteins in Jurkat T-cells. Cells were incubated with 10 μg ml^−1^ of anti-CD3 for the indicated times before lysates. (**c**) Co-immunoprecipitation analysis of Gα_q/11_ binding to CD3ɛ and PLCβ3 in control and ORP4L knockdown cells. Cells were infected with shNT or shORP4L lentivirus for 72 h, and then incubated with or without 10 μg ml^−1^ of anti-CD3 for 5 min before lysates. (**d**) Co-immunoprecipitation analysis of Gα_q/11_ binding to CD3ɛ and PLCβ3 in Jurkat T-cell lysates before immunodepletion with ORP4L antibody. (**e**) Co-immunoprecipitation analysis Gα_q/11_ binding to CD3ɛ and PLCβ3 in normal T-cells. Cells were incubated with or without 10 μg ml^−1^ of anti-CD3 for 10 min before lysates. (**f**) Confocal immunofluorescence microscopy analysis of CD3ɛ (green), ORP4L (red) and pan-Cadherin (blue) localization in shNT and shORP4L transduced Jurkat T-cells. Scale bars, 10 μm. (**g**) Confocal immunofluorescence microscopy analysis of Gα_q/11_ (green), ORP4L (red) and pan-Cadherin (blue) localization in shNT and shORP4L transduced Jurkat T-cells. Scale bars, 10 μm. (**h**) Confocal immunofluorescence microscopy analysis of PLCβ3 (green), ORP4L (red) and pan-Cadherin (blue) localization in shNT and shORP4L transduced Jurkat T-cells. Scale bars, 10 μm. (**i**–**k**) Confocal microscopy analysis of CD3ɛ (blue), Gα_q/11_ (green) and p–PLCβ3 (red) localization in shNT and shORP4L transduced Jurkat T-cells (**i**), Molt-4 cell (**j**) and primary T-ALL cells (**k**). Scale bars, 10 μm. For the confocal immunofluorescence above, cells were infected with shNT or shORP4L lentivirus for 72 h and then stimulated before staining for 5 min with or without 10 μg ml^−1^ anti-CD3.

To address the mechanistic role of ORP4L in the energy homeostasis of T-ALL cells, we carried out a proteomic analysis of ORP4L-interacting components in Jurkat T-cells with an antibody specific for ORP4L. Anti-ORP4L and control IgG immunoprecipitates of cells stimulated with anti-CD3 were separated on SDS–PAGE (Fig. 2a), and polypeptides specifically associated with ORP4L were identified by mass spectrometry. A total of 14 proteins were identified as potential ORP4L binding partners by subtracting proteins precipitated by control IgG from those identified in anti-ORP4L precipitated specimens (Supplementary Table 2). CD3ɛ, Gα_q**/11**_ and PLCβ3 were among these candidates; the finding was confirmed by western blot analysis of the immunoprecipitates (Fig. 2a). Binding of Gα_q/**11**_ to CD3ɛ is activated upon anti-CD3 stimulation^28^, and these proteins can associate with PLCβ for signal transduction^29^,^30^. Physical interactions between ORP4L and its binding partners were further investigated by co-immunoprecipitation. In the absence of anti-CD3 treatment, low levels of complexes of CD3ɛ and PLCβ3 were detected. On anti-CD3 stimulation, interaction of ORP4L with these two proteins increased in a time-dependent manner, but no difference was observed in the association of ORP4L and Gα_q/11_ (Fig. 2b). The interactions between ORP4L, CD3ɛ, Gα_q/11_ and PLCβ3 raised the possibility that ORP4L could be required for the CD3ɛ–Gαq_q/11_ or Gα_q/11_–PLCβ3 interactions. To test this hypothesis, we performed co-immunoprecipitation with anti-Gα_q/11_ in Jurkat T-cells with ORP4L knockdown. Gα_q/11_ was bound to CD3ɛ and PLCβ3 upon anti-CD3 stimulation, but the binding was reduced in ORP4L knockdown cells (Fig. 2c). Moreover, immunodepletion/immunoprecipitation experiments revealed that ORP4L depletion markedly reduced the CD3ɛ–Gα_q/11_ and Gα_q/11_–PLCβ3 interactions (Fig. 2d). Normal T-cells exhibited no detectable ORP4L protein expression, and Gα_q/11_ failed to bind to CD3ɛ and PLCβ3 upon anti-CD3 stimulation of these cells (Fig. 2e). The above results strongly suggest that ORP4L acts as an adaptor that couples CD3ɛ, Gα_q/11_ and PLCβ3 upon anti-CD3 stimulation.

Confocal imaging analyses showed that CD3ɛ clustered in a non-uniform manner at the plasma membrane of unstimulated Jurkat T-cells. Anti-CD3 stimulation, however, induced a striking redistribution of CD3ɛ in the plasma membrane and increased its co-localization with ORP4L (Fig. 2f). In contrast, redistribution of CD3ɛ was not observed in ORP4L-depleted cells (Fig. 2f). Gα_q/11_ was distributed uniformly at the plasma membrane and co-localized with ORP4L; its location did not change upon anti-CD3 stimulation or ORP4L depletion (Fig. 2g). PLCβ3 exhibited an intranuclear localization in unstimulated cells (Fig. 2h; Supplementary Fig. 4a). Anti-CD3 stimulation induced a partial but clear translocation of PLCβ3 to the plasma membrane where it co-localized with ORP4L (Fig. 2h). Cell fractionation followed by the western blot analysis provided further evidence for translocation of PLCβ3 from the nucleus to the plasma membrane upon anti-CD3 stimulation (Supplementary Fig. 4b). Immunofluorescence experiments revealed localization of ORP4L at the plasma membrane, but most of this protein remained in the soluble cytosolic fraction after cell fractionation. To clarify the reason for this contradiction, we treated cells before lysis with membrane permeable cross-linking agent dithiobis-succinimidyl propionate (DSP). The intensity of the ORP4L band in plasma membrane fraction increased substantially after DSP treatment (Supplementary Fig. 4c), consistent with the view that ORP4L associates with plasma membrane via peripheral, weak or transient interactions and that the lysis procedure disrupts the association. Of note, ORP4L knockdown markedly inhibited the PLCβ3 translocation upon anti-CD3 stimulation (Fig. 2h). The responding cell number and the quantity of translocated PLCβ3 protein were reduced in ORP4L knockdown cells (Supplementary Fig. 4d), which could be rescued by ORP4L overexpression (Supplementary Fig. 4e). Confocal imaging showed that the active phosphorylated form of PLCβ3 (p-PLCβ3)^31^,^32^ was present only in the plasma membrane but not in the nucleus of anti-CD3 stimulated cells (Supplementary Fig. 4f), indicating that intranuclear PLCβ3 was inactive. Triple staining for CD3ɛ, Gα_q/11_ and p-PLCβ3 displayed a high degree of co-localization, consistent with the notion that these proteins are assembled into complexes upon anti-CD3 stimulation in Jurkat (Fig. 2i), Molt-4 (Fig. 2j) and primary T-ALL cells (Fig. 2k). Whereas, the co-localization was inhibited in cells subjected to ORP4L knockdown. To conclude, the present results thus suggest that ORP4L functions as an adaptor/scaffold for the assembly of CD3ɛ, Gα_q/11_ and PLCβ3 into a signalling complex.

### ORP4L mediates PLCβ3 activation and IP_3_ production

**Figure 3:**
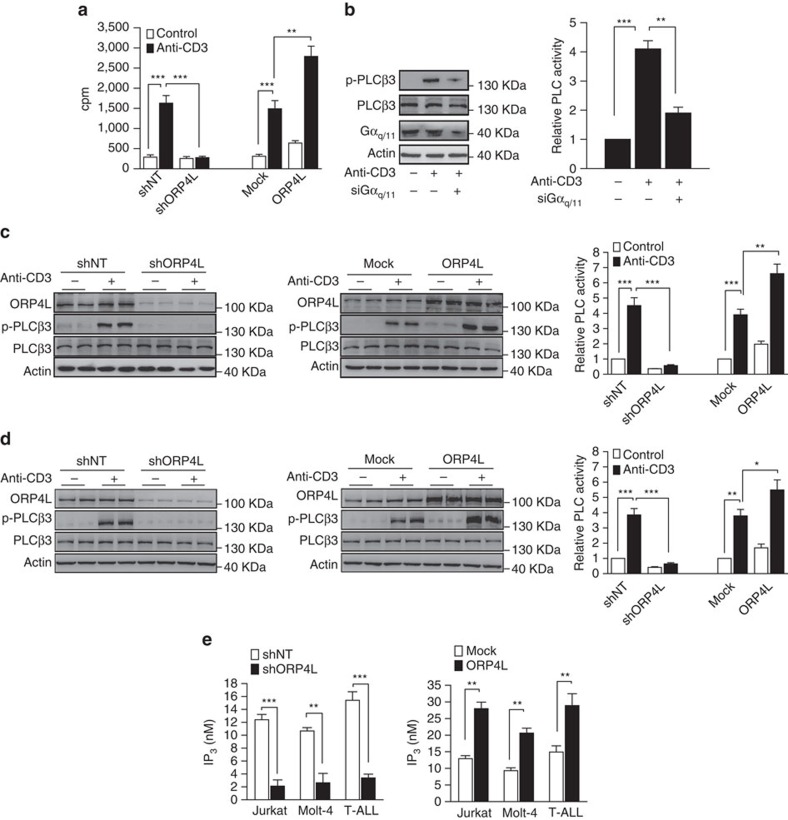
ORP4L is required for PLCβ3 activation. (**a**) Specific [^35^S]GTPγS exchange in Gα_q/11_ in Jurkat T-cells with ORP4L knockdown or overexpression. Cells were stimulated with or without 10 μg ml^−1^ anti-CD3 for 5 min before analysis. (**b**) PLCβ3 phosphorylation and PLC activity in Jurkat T-cells with Gα_q/11_ knockdown induced with or without 10 μg ml^−1^ anti-CD3 for 5 min. (**c**,**d**) PLCβ3 phosphorylation and PLC activity in Jurkat T-cells (**c**) and Molt-4 cells (**d**) with ORP4L knockdown or overexpression. Cells were stimulated with 10 μg ml^−1^ anti-CD3 for 5 min. (**e**) IP_3_ production in Jurkat T-cells, Molt-4 cells and primary T-ALL cells (*n*=3) with ORP4L knockdown (left) or overexpression (right). Cells were stimulated with 10 μg ml^−1^ anti-CD3 for 5 min. The data represent mean±s.d. value from an experiment performed in triplicate, **P<0.01, ***P<0.001, Student's *t* test.

Functional relationships among the signalling complex components scaffolded by ORP4L were further interrogated. In Jurkat T-cells, anti-CD3 stimulation resulted in GTP exchange within Gα_q/11_ (Fig. 3a), indicating the activation of this protein. ORP4L depletion inhibited the Gα_q/11_ activation, while its overexpression facilitated this activation (Fig. 3a). Moreover, phosphorylation of PLCβ3 and PLC activity upon anti-CD3 stimulation were abolished by Gα_q/11_ knockdown (Fig. 3b), indicating that PLCβ3 is under these conditions activated by Gα_q/11_. ORP4L knockdown decreased, whereas ORP4L overexpression increased p–PLCβ3 protein levels and PLC activity in Jurkat T-cells (Fig. 3c) and Molt-4 cells (Fig. 3d). These results demonstrate that PLCβ3 is activated in T-ALL cells by Gα_q/11_, which requires the presence of ORP4L.

The prominent role of PLCβ3 in T-ALL cells was further clarified. PLCγ1 knockdown decreased IP_3_ production and intracellular Ca^2+^ [Ca^2+^]_i_ peak amplitude in normal T-cells upon anti-CD3 stimulation (Supplementary Fig. 5a), but no significant change was observed in primary T-ALL cells (Supplementary Fig. 5b) or Jurkat T-cells (Supplementary Fig. 5c). By contrast, PLCβ3 knockdown resulted in a significant reduction of IP_3_ production and [Ca^2+^]_i_ peak amplitude in primary T-ALL (Supplementary Fig. 5b) and Jurkat T-cells (Supplementary Fig. 5c), while no such effect was observed in normal T-cells (Supplementary Fig. 5a). Overexpression of PLCβ3, but not PLCγ1, increased IP_3_ production and amplified [Ca^2+^]_i_ peak amplitude in Jurkat T-cells (Supplementary Fig. 5d). These findings suggest that PLCγ1 is essential for IP_3_ production and Ca^2+^ release in normal T-cells, whereas PLCβ3 is a major regulator of these responses in T-ALL cells.

ORP4L knockdown significantly reduced IP_3_ production in Jurkat T-cells, Molt-4 cells and primary T-ALL cells (Fig. 3e, left), while its overexpression significantly increased IP_3_ production (Fig. 3e, right). Furthermore, overexpression of ORP4L rescued the IP_3_ level in ORP4L knockdown cells (Supplementary Fig. 6a), and the increased IP_3_ production upon overexpression of ORP4L was abrogated upon PLCβ3 silencing (Supplementary Fig. 6b). Also overexpression of PLCβ3 increased the IP_3_ production, an effect that was abolished upon ORP4L knockdown (Supplementary Fig. 6c). Exogenous PIP_2_ can increase IP_3_ production in a dose-dependent manner (Supplementary Fig. 6d, left panel). ORP4L depletion attenuated, while ORP4L overexpression increased the PIP_2_-induced IP_3_ production (Supplementary Fig. 6d, middle and right panels). Altogether, these findings strongly suggest that ORP4L regulates cellular IP_3_ production.

### ORP4L modulates Ca^2+^ signalling in T-ALL cells

**Figure 4:**
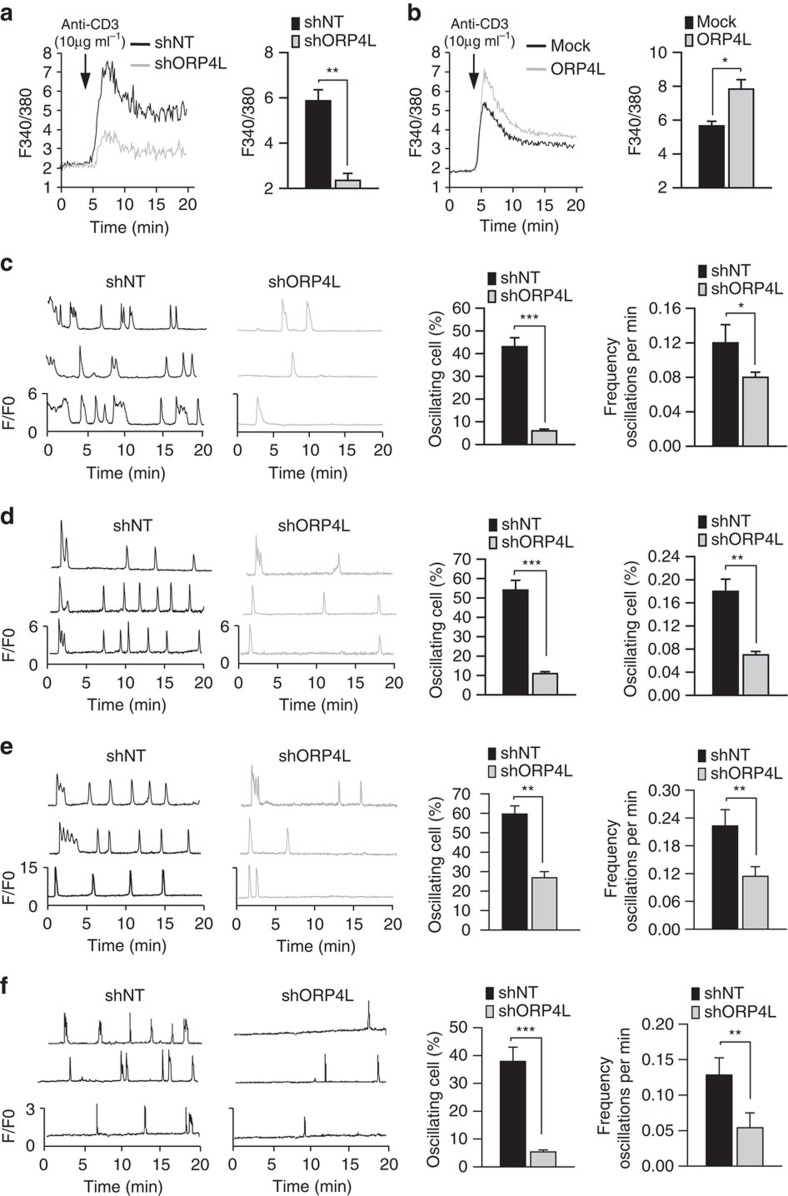
ORP4L modulates Ca^2+^ signalling in T-ALL cells. (**a**,**b**) Jurkat T-cells subjected to ORP4L knockdown (**a**) or overexpression (**b**) were stimulated with 10 μg ml^−1^ anti-CD3. Changes in [Ca^2+^]_i_ were recorded as the F340/380 ratio using Fura-2 AM. Average [Ca^2+^]_i_ responses and quantification of [Ca^2+^]_i_ peak amplitudes are shown. (**c**–**e**) Spontaneous [Ca^2+^]_i_ oscillations in Jurkat T-cells (**c**), Molt-4 cells (**d**) and primary T-ALL cells (**e**) with ORP4L knockdown. Changes in [Ca^2+^]_i_ were recorded as F/F_0_ ratio using Fluo-4-AM in confocal Ca^2+^ imaging. Spontaneous [Ca^2+^]_i_ oscillations in three representative cells (left) were measured, and the difference in frequency and number of oscillating cells is shown (right). (**f**) Spontaneous [Ca^2+^]_m_ oscillations in Jurkat T-cells with ORP4L knockdown. Changes in [Ca^2+^]_m_ were recorded as *F*/*F*_0_ ratio using Rhod-2-AM in confocal Ca^2+^ imaging. For the Jurkat T-cells the data represent mean±s.d. value from an experiment performed in triplicate, and for primary T-ALL cells the mean±s.d. value of *n*=3 primary T-ALL specimens. *P<0.05, **P<0.01, ***P<0.001, Student's *t* test.

The function of ORP4L was further explored by assaying Ca^2+^ release in the cytosol. In agreement with the observed in IP_3_ production, ORP4L knockdown markedly reduced the [Ca^2+^]_i_ peak amplitude (Fig. 4a), whereas ORP4L overexpression enhanced the amplitude in anti-CD3 stimulated Jurkat T-cells (Fig. 4b). The similar results were observed in a Ca^2+^-free medium (Supplementary Fig. 7a,b). ORP4L manipulations had no effect on Ca^2+^ influx (Supplementary Fig. 7a,b), supporting the view that Ca^2+^ released upon ORP4L manipulation originates from the ER. Consistently, direct measurement of the ER Ca^2+^ revealed that in ORP4L knockdown cells the amplitude of ER Ca^2+^ depletion upon anti-CD3 stimulation was smaller than in controls, whereas ORP4L overexpression increased the depletion amplitude (Supplementary Fig. 7c,d). Thapsigargin (TG) -induced Ca^2+^ elevation is an indirect measure of ER Ca^2+^ content^33^. We found no difference in the magnitude of the TG releasable Ca^2+^ pool in control and ORP4L knockdown or overexpressing cells (Supplementary Fig. 7e,f). Moreover, the ORP4L manipulations did not affect the total amount of intracellular Ca^2+^ (Supplementary Fig. 7g). These results further support the conclusion that ORP4L regulates Ca^2+^ release from the ER.

Single cell confocal imaging of spontaneous [Ca^2+^]_i_ oscillations revealed transient [Ca^2+^]_i_ spikes in 43% of control Jurkat T-cells. In contrast, spontaneous [Ca^2+^]_i_ spikes were observed in only 6% of ORP4L knockdown cells and at a lower frequency than in controls (0.08 versus 0.12 oscillations min^−1^) (Fig. 4c). Similar results were consistently obtained in Molt-4 cells (Fig. 4d) and primary T-ALL cells (Fig. 4e). The pulsatile release of Ca^2+^ underlying [Ca^2+^]_i_ oscillations is transmitted efficiently into the mitochondrial matrix, giving rise to associated oscillations of [Ca^2+^]_m_. ORP4L knockdown also reduced oscillations of [Ca^2+^]_m_ in Jurkat T-cells (Fig. 4f).

ER–mitochondrial contact sites are known to mediate Ca^2+^ transfer from ER to mitochondria^34^,^35^. We employed transmission electron microscopy and confocal microscopy to investigate the possibility that ORP4L could modify the ER–mitochondrial contact sites. The results failed to reveal a change in the quantity of ER–mitochondrial contacts in ORP4L knockdown cells (Supplementary Fig. 8a,b). Cholesterol is a critical component of biological membranes and has an essential role in mitochondrial fuctions^36^. Considering that ORP4L is a sterol-binding protein, we analyzed the effect of ORP4L silencing on cholesterol transport from the plasma membrane to ER and mitochondria using fluorescent BODIPY-cholesterol. After 1-h chase of the BODIPY-cholesterol label inserted in plasma membranes, no difference was observed between control and ORP4L knockdown cells in the transport of the tracer to either target organelle (Supplementary Fig. 9a,b). The present results thus suggest that ORP4L increases the release of Ca^2+^ from the ER but most likely does not control ER–mitochondrial contacts or cholesterol transport to these compartments.

The *OSBP2* gene encodes ORP4L as well as two truncated variants ORP4M and ORP4S^18^. Similar to ORP4L, ORP4M and ORP4S were detected in primary T-ALL specimens and T-ALL cell lines but not in normal T-cells as analyzed by reverse transcription-PCR (RT-PCR) (Supplementary Fig. 10a). To determine the role of ORP4M, ORP4S, the PH domain and the FFAT motif of ORP4 in the proposed pathway, we overexpressed a series of constructs in Jurkat T-cells (Supplementary Fig. 10b), followed by analysis of IP_3_ production and amplitude of Ca^2+^ release upon anti-CD3 stimulation. The results showed that ORP4M, ORP4S, the truncated fragment lacking the PH domain and the PH domain alone were unable to increase the IP_3_ production (Supplementary Fig. 10c) and the Ca^2+^ release (Supplementary Fig. 10d). However, the mutant with the FFAT motif inactivated retained the activities of ORP4L. These results indicated that the ORP4L PH domain is essential for the proposed pathway, while FFAT motif is not absolutely required.

### ORP4L sustains Ca^2+^-based bioenergetics in T-ALL cells

**Figure 5:**
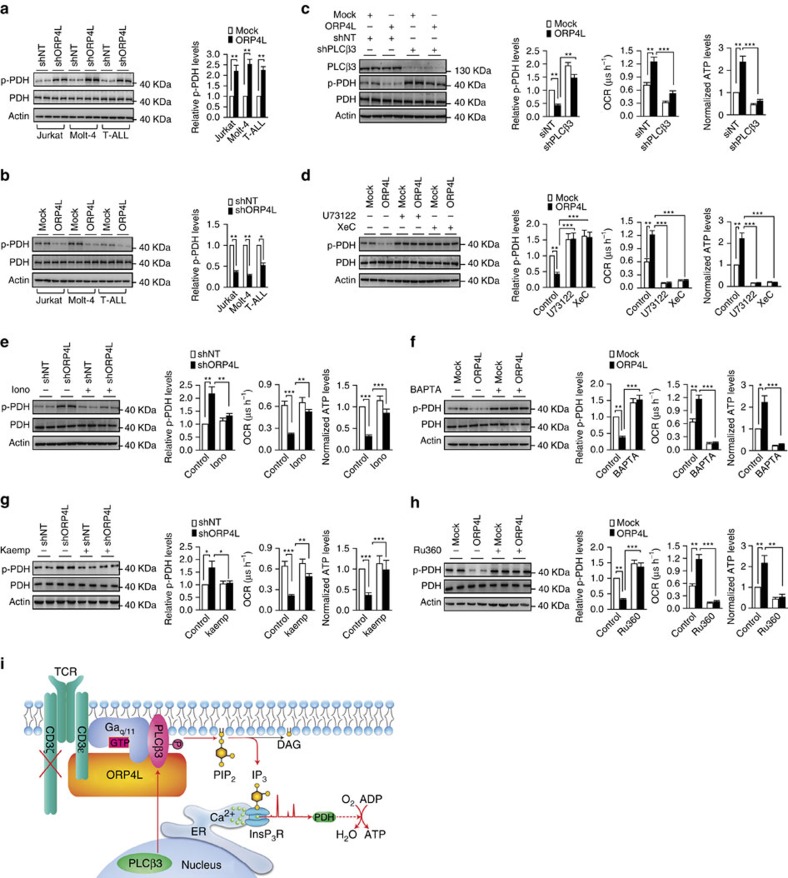
ORP4L sustains Ca^2+^-dependent bioenergetics in T-ALL cells. (**a**,**b**) Western blot analysis of PDH activation in Jurkat, Molt-4 and primary T-ALL cells with ORP4L knockdown (**a**) and overexpression (**b**). p-PDH/PDH expressed as fold change over control. (**c**) PDH activation (left), OCR (middle) and ATP levels (right) in Jurkat T-cells with ORP4L knockdown alone or in combination with shPLCβ3. (**d**) PDH activation (left), OCR (middle) and ATP levels (right) in control or ORP4L overexpressing Jurkat T-cells treated with or without U73122 (5 μM for 1 h) or XeC (2 μM for 1 h). (**e**) PDH activation (left), OCR (middle) and ATP levels (right) in control or ORP4L knockdown Jurkat T-cells treated with or without ionomycin (2 mg l^−1^ for 1 h). (**f**) PDH activation (left), OCR (middle) and ATP levels (right) in control or ORP4L overexpressing Jurkat T-cells treated with or without BAPTA-AM (50 μM for 1 h). (**g**) PDH activation (left), OCR (middle) and ATP levels (right) in control or ORP4L knockdown Jurkat T-cells treated with or without MCU agonist, kaempferol (2 μM, 30 min). (**h**) PDH activation (left), OCR (middle) and ATP levels (right) in control or ORP4L overexpressing Jurkat T-cells treated with or without MCU inhibitor, RU360 (5 μM, 30 min). The data represent mean±s.d. value from an experiment performed in triplicate. *P<0.05, **P<0.01, ***P<0.001, Student's *t* test. (**i**) Model outlining the functions of ORP4L. In T-ALL cells, CD3ζ chain expression is defective, and ORP4L couples CD3ɛ to PLCβ3/Gα_q/11_ to control the relocation and activation of PLCβ3, which leads to increased IP_3_ generation and Ca^2+^ release from ER required for sustaining of cell bioenergetics.

Ca^2+^ oscillations released periodically from the ER are taken up by mitochondria via the MCU^8^, which stimulates mitochondrial dehydrogenases required for the maintenance of cell bioenergetics^9^,^10^. Dephosphorylation of mitochondrial pyruvate dehydrogenase (PDH) by a Ca^2+^-dependent phosphatase enhances its activity^9^. PDH was hyperphosphorylated in Jurkat, Molt-4 and primary T-ALL cells with ORP4L knockdown (Fig. 5a), whereas ORP4L overexpression reduced PDH phosphorylation (Fig. 5b). To strengthen the link between ORP4L, Ca^2+^signalling and mitochondrial metabolism, we analyzed PDH phosphorylation, OCR and ATP levels in ORP4L manipulated Jurkat T-cells subjected to PLCβ3 knockdown or a series of inhibitor/agonist treatments. PDH dephosphorylation, OCR and ATP production increase induced by ORP4L overexpression were abolished by PLCβ3 knockdown (Fig. 5c), by the PLC inhibitor U73122 and the InsP_3_R inhibitor xestospongin C (XeC) (Fig. 5d). The effects of ORP4L knockdown on increased PDH phosphorylation, reduced OCR and ATP levels were rescued by the Ca^2+^ transporter ionomycin (Fig. 5e) and the MCU agonist kaempferol^37^ (Fig. 5g). By contrast, PDH dephosphorylation and increased OCR and ATP levels upon ORP4L overexpression could be abolished by the Ca^2+^ chelating agent BAPTA (Fig. 5f) and the MCU inhibitor RU360 (Fig. 5h). Energy produced during mitochondrial respiration is maintained as a high membrane potential^38^, which is mediated through a Ca^2+^-dependent mechanism^39^. ORP4L knockdown significantly reduced the mitochondrial membrane potential and NAD(P)H autofluorescence (Supplementary Fig. 11a,b). These findings suggest that ORP4L facilitates via stimulation of IP_3_ production and ER Ca^2+^ release the delivery of Ca^2+^ to mitochondria and thereby regulates the phosphorylation of PDH to maintain mitochondrial bioenergetics (Fig. 5i).

It was obviously of interest to study whether forced expression of ORP4L in normal T-cells would be sufficient to divert their energy metabolism toward robust oxidative phosphorylation. After transfecting ORP4L into normal T-cells (Supplementary Fig. 12a), we analyzed IP_3_ production, OCR and ATP production, but found no significant difference between control and ORP4L overexpressing cells (Supplementary Fig. 12b). These findings suggest that, although ORP4L is necessary for the reprogramming of T-ALL cell bioenergetics, it is alone not sufficient to induce the process when overexpressed acutely.

### ORP4L is essential for T-ALL cell survival *in vitro* and *in vivo*

**Figure 6:**
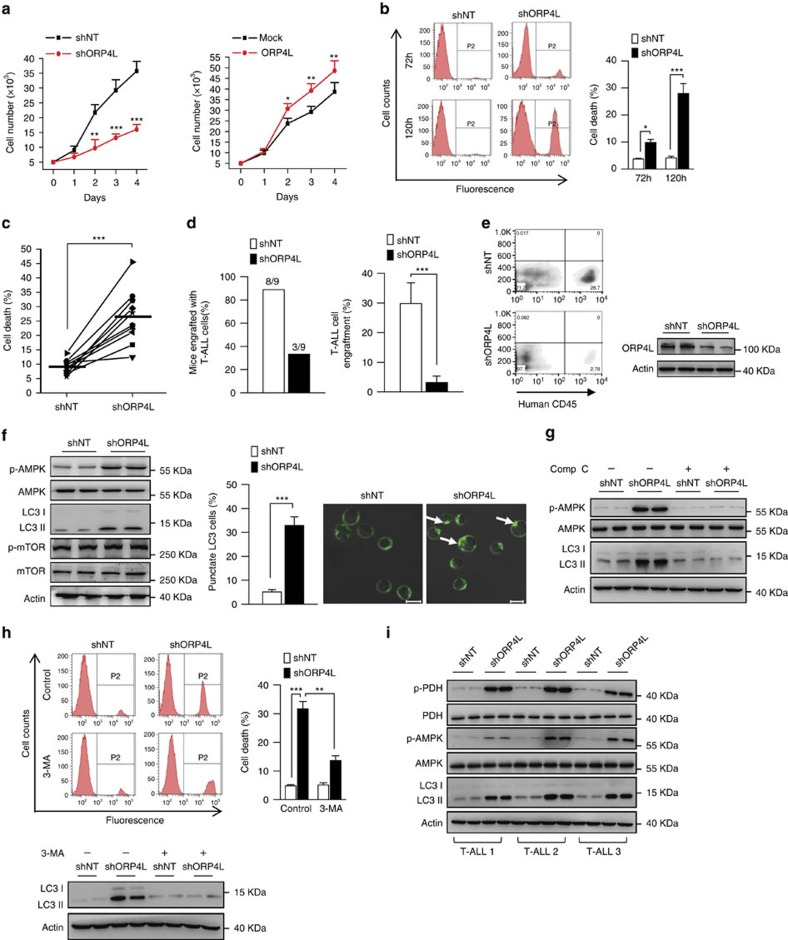
ORP4L is essential for T-ALL cell survival. (**a**) Proliferation assay in Jurkat T-cells with ORP4L knockdown (left) or overexpression (right). (**b**) Cell death analysis in Jurkat T-cells with ORP4L knockdown for 72 and 120 h. (**c**) Cell death analysis in primary T-ALL cells from 10 specimens with ORP4L knockdown for 120 h. (**d**) The proportion of mice engrafted with primary T-ALL cells (left), and engrafted primary T-ALL cells as a percentage of the total white blood cells (right), (*n*=9 of shNT mice, *n*=3 of shORP4L mice). (**e**) Flow cytometric analysis of engrafted primary T-ALL cells in the blood of NOD/SCID mice with representative dot plots are shown. Western blot indicates ORP4L knockdown efficiency of T-ALL cells after infected with lentivirus for 72 h. (**f**) Western blot analysis of p-AMPK, LC3 and p-mTOR in control and ORP4L knockdown Jurkat T-cells (left). Confocal images of LC3 puncta in control and ORP4L knockdown Jurkat T-cells (middle and right). Arrows show LC3 puncta (200 cells from three experiments with 10 random fields/experiment). Scale bars, 10 μm. (**g**) Western blot of p-AMPK and LC3 in control and ORP4L knockdown Jurkat T-cells with or without AMPK inhibitor compound C (comp C, 15 μM, 4 h). (**h**) Cell death analysis in Jurkat T-cells with or without autophagy inhibitor, 3-MA. After infection with shNT or shORP4L lentivirus for 48 h, cells were incubated with or without 5 mM 3-MA for further 72 h, and then used for cell death analysis. The Western blot at the bottom verifies that 3-MA inhibited autophagy formation. (**i**) Western blot of p-PDH, p-AMPK and LC3 in primary T-ALL cells with ORP4L knockdown. The data represent mean±s.d. value from an experiment performed in triplicate. *P<0.05, **P<0.01, ***P<0.001, Student's *t* test.

In studies assessing the overall role of ORP4L in T-ALL cells, we found that ORP4L expression was positively correlated with Jurkat T-cell proliferation (Fig. 6a). ORP4L knockdown strongly accelerated Jurkat T-cells (Fig. 6b) and primary T-ALL cells death (Fig. 6c). A xenograft model was established in NOD/SCID mice to determine the contribution of ORP4L to T-ALL cell survival *in vivo*. Mice were injected with primary T-ALL cells infected with either shORP4L- or control shNT-lentivirus. At 2 weeks post-injection engraftment had occurred in eight of nine mice injected with shNT cells but only in three of nine of mice receiving shORP4L cells (Fig. 6d, left panel). Moreover, the percentage of engrafted cells was markedly lower in mice injected with shORP4L cells (Fig. 6d, right panel and Fig. 6e).

Defective Ca^2+^ transfer from the ER to the mitochondria results in activation of the cellular energy sensor AMP kinase (AMPK), which induces autophagy^9^. To understand the pathway of cell death upon ORP4L knockdown, we analyzed the phosphorylation/activation of AMPK, and found that phospho-AMPK (p-AMPK) significantly increased upon ORP4L knockdown (Fig. 6f). Also, elevated autophagy upon ORP4L knockdown was detected by measurements of the autophagy marker LC3 II as well as increased LC3 puncta observed by immunofluorescence microscopy (Fig. 6f). Enhanced AMPK activity can in certain cases induce autophagy by inhibition of mTOR^40^. Nevertheless, mTOR phosphorylation at Ser2448 was similar in control and ORP4L knockdown cells (Fig. 6f), indicating that AMPK activation, but not inhibition of mTOR, is required for enhanced autophagy induced in the absence of ORP4L.

Autophagy can either suppress or support the growth of cancer cells depending on the cellular context^2^. Autophagy in the ORP4L knockdown cells was inhibited by the AMPK inhibitor compound C (Fig. 6g), indicating that AMPK is upstream of the autophagy induction in ORP4L knockdown cells. The death of these cells was markedly reduced by the inhibitor of autophagy, 3-MA (Fig. 6h). Consistent with the results in Jurkat T-cells, ORP4L knockdown activated PDH and AMPK, and enhanced autophagy also in primary T-ALL cells (Fig. 6i).

## Discussion

We confirmed herein that, in contrast to normal T-cells, T-ALL cells preferentially utilize robust oxidative phosphorylation as a source of ATP, similar to a recent observation in chronic lymphocytic leukemia cells^38^ and leukemia stem cells^41^. Our novel finding is that PLCβ3, activated through G protein-dependent signal transduction, serves as a major enzyme for IP_3_ generation and intracellular Ca^2+^ and energy homeostasis in T-ALL cells, due to a selective up-regulation of ORP4L in malignant transformed T-cells.

Upon analysis of the underlying molecular mechanisms, we found that ORP4L specifically interacts with CD3ɛ, Gα_q/11_ and PLCβ3. Anti-CD3 stimulation enhanced these interactions, suggesting assembly of a signalling complex upon anti-CD3 stimulation. Immunodepletion/co-immunoprecipitation assays showed that the binding of CD3ɛ to Gα_q/11_, crucial for PLCβ3 activation, depends on ORP4L. In addition, confocal imaging showed CD3ɛ and PLCβ3 redistribution and co-localization with Gα_q/11_ only in the presence of ORP4L. Taken together, these findings suggest that ORP4L acts as an adaptor/scaffold to recruit CD3ɛ, Gα_q/11_ and PLCβ3 for the formation of a G protein-dependent signalling complex in T-ALL cells.

PLCs perform a catalytic function at the plasma membrane where their substrate, PIP_2_, is localized. However, PLCs also exist at other subcellular locations such as the cytoplasm and the nucleus^42^,^43^. This separation of an enzyme from its substrates represents an important regulatory mechanism, but understanding the molecular interactions and subcellular targeting events that control PLC function requires new insight. We demonstrate that PLCβ3 mainly exists within the nucleus of T-ALL cells and translocates to the plasma membrane upon stimulation. Therefore, we envision that a pathway contributing to signal transduction from CD3 to the nucleus may be affected by ORP4L, and our new findings provide insight into points at which this pathway is regulated. In particular, ORP4L enables CD3 signalling to Gα_q/11_. It also facilitates the shift of PLCβ3 location from the nucleus to the plasma membrane. This promotes assembly of CD3ɛ, Gα_q/11_ and PLCβ3 into a macromolecular complex to enhance the efficiency, selectivity and specificity of CD3-G protein signal transduction for Ca^2+^ homeostasis in T-ALL cells.

In normal T-cells subjected to anti-CD3 stimulation, LAT (pp36) is heavily tyrosine phosphorylated and subsequently binds PLCγ1 to increase IP_3_ and intracellular Ca^2+^ in a G protein-independent manner^44^,^45^,^46^. This process is required for CD3ζ chain recruitment to active ZAP70 with a central role in CD3 signaling^47^,^48^. However, CD3ζ chain expression in T-ALL cells is defective^49^. Thus, CD3 signalling is swapped to Gα_q/11_ in the presence of ORP4L and sequentially activates Gα_q/11_ and PLCβ3, which becomes the dominant enzyme for IP_3_ generation and intracellular Ca^2+^ homeostasis in T-ALL cells. We conclude that ORP4L in this way supports mitochondrial oxidative phosphorylation for T-ALL cell survival (Fig. 5i).

In agreement with the observed effects of ORP4L on IP_3_ generation, spontaneous [Ca^2+^]_i_ oscillations in the cytosol and the mitochondria were reduced in ORP4L knockdown cells. Furthermore, ORP4L manipulations were shown to modify the mitochondrial PHD activity, OCR and ATP levels. These observations provide evidence that ORP4L controls ER Ca^2+^ release by facilitating the catalytic action of PLCβ3 to sustain PHD activity and ATP production. In contrast to many other types of cancers, T-ALL cells do not appear to over-utilize aerobic glycolysis, also known as the Warburg effect^24^, but rely on oxidative phosphorylation for ATP production. The results in our study and those recently reported in chronic lymphocytic leukemia cells^38^ and leukemia stem cells^41^ reveal a defect in employing glycolysis. The reasons for this phenotype represent an important avenue of future investigation. Enhanced oxidative phosphorylation induced by reprogramming of Ca^2+^ homeostasis by ORP4L in T-ALL cells offers a plausible mechanistic explanation for the mode of energy metabolism in these transformed leukemia cells.

In accordance with the role in ATP production, ORP4L acts as a key protein in T-ALL cell survival. ORP4L-depleted cells displayed growth suppression and were sensitized to energy status-dependent autophagic cell death *in vitro*. Further support for this concept was produced by primary T-ALL cell engraftment in a NOD/SCID model. Reduced engraftment of T-ALL cells with ORP4L knockdown in a NOD/SCID model demonstrated that ORP4L is crucial for T-ALL cell survival *in vivo*.

In conclusion, our results reveal that selective ORP4L expression in T-ALL cells mediates G protein-dependent signalling and leads to translocation and activation of PLCβ3 to maintain Ca^2+^ homeostasis and bioenergetics. ORP4L is thus essential for T-ALL cell survival and could therefore be a viable therapeutic target for T-ALL. Inhibition of its expression/activity by RNAi or small-molecular compounds may serve as a new approach to treat this deadly malignancy.

## Methods

### Reagents and antibodies

Fura-2-AM, Fluo-4-AM, Rhod-2-AM, Fluo-5N-AM, BODIPY-cholesterol, TRIzol reagent, Alexa Fluor-488 goat anti-mouse IgG (Catalogue No. A-11001, diluted 1:200 for immunofluorescence), Alexa Fluor-543 goat anti-rabbit IgG (Catalogue No. A-11035, diluted 1:200 for immunofluorescence) and Alexa Fluor-647 donkey anti-goat IgG (Catalogue No. A-21447, diluted 1:200 for immunofluorescence) were purchased from Invitrogen (Carlsbad, CA, USA). Hoechst 33342, U73122, XeC, thapsigargin and JC-1 were from Sigma-Aldrich (St Louis, MO, USA). Compounds C, Ru360 and ionomycin were from Merck Millipore (Billerica, MA, USA). Kaempferol and BAPTA-AM were purchased from Selleckchem. LEAF purified human anti-CD3 (clone HIT3a, Catalogue No. 300314), Alexa Fluor-647 anti-human CD45 (Catalogue No. 304056, diluted 1:20 for flow cytometric) and anti-H2A (Catalogue No. 613301, diluted 1:500 for immunoblotting) were obtained from BioLegend (San Diego, CA, USA). Anti-PLCβ3 (Catalogue No. sc-133231, diluted 1:200 for immunoblotting and 1:50 for immunofluorescence), anti-PLCγ1 (Catalogue No. sc-7290, diluted 1:200 for immunoblotting), anti-Gα_q/11_ (Catalogue No. sc-392, diluted 1:200 for immunoblotting and 1:50 for immunofluorescence), anti-pan-cadherin (Catalogue No. sc-1499, diluted 1:200 for immunoblotting and 1:50 for immunofluorescence) and anti-PTEN (Catalogue No. sc-7974, diluted 1:200 for immunoblotting) were from Santa Cruz (Santa Cruz, CA, USA). Anti-p-PDH (Catalogue No. NB110–93479, diluted 1:1,000 for immunoblotting) was obtained from Novus (St Louis, MO, USA); Anti-p–PLCβ3 (Catalogue No. 2481, diluted 1:1,000 for immunoblotting and 1:100 for immunofluorescence), anti-PDH (Catalogue No. 2784, diluted 1:1,000 for immunoblotting), anti-mTOR1(Catalogue No. 2972, diluted 1:1,000 for immunoblotting), anti-p-mTOR1 (Ser2448) (Catalogue No. 2971, diluted 1:1,000 for immunoblotting), anti-Notch-1 (Catalogue No. 3268, diluted 1:1,000 for immunoblotting), anti-AMPK (Catalogue No. 2603, diluted 1:1,000 for immunoblotting) and anti-p-AMPK (Thr172) (Catalogue No. 2535, diluted 1:1,000 for immunoblotting) from Cell Signaling (Beverly, MA, USA), and anti-actin (Catalogue No. 60008-1, diluted 1:3,000 for immunoblotting) was from Proteintech Group (Chicago, IL). Anti-ORP4L (Catalogue No. HAP021514, diluted 1:1,000 for immunoblotting and 1:200 for immunofluorescence) from Sigma-Aldrich was used for all of the experiments except for ORP4L-associated proteome analysis. Cross-linking reagent DSP was purchased from Thermo Scientific.

### cDNA constructs

Human full length ORP4L, ORP4M, ORP4S, PLCβ3 and PLCγ1 cDNAs were amplified by PCR amplification from HeLa cell cDNA, subcloned into pcDNA4HisMaxC (Invitrogen) vector. The PH domain and FFAT motif deletion fragments were amplified by PCR from ORP4L cDNA. Primers used for these constructs can be found in Supplementary Table 4. The construct were verified by sequencing.

### Human leukocyte specimens and cell lines

This study was approved by the institutional ethics committee of Jinan University and was performed in accordance with the Declaration of Helsinki. Fresh leukocytes were isolated from peripheral blood of healthy human donors and T-ALL patients after obtaining written informed consent. The clinical information and application of total of 18 T-ALL samples are provided in Supplementary Table 1. Naive CD3^+^ T-cells were isolated using an Enhanced Human T Cell Recovery Column Kit (Cedarlane, Burligton, ON, Canada) according to the manufacturer's instructions and maintained in RPMI 1640 containing 10% FBS. Jurkat, Molt-4, CEM and MT-4 cells were purchased from American Type Culture Collection and maintained in RPMI 1640 containing 10% FBS, 100 U ml^−1^ penicillin, and 100 μg ml^−1^ streptomycin at 37 °C in a humidified incubator with 5% CO_2_. All of cell lines were authenticated by Promega short-tandem repeat analysis and tested for mycoplasma contamination before experiments.

### Gene transfer

For ORP4L, PLCβ3 and PLCγ1 knockdown and ORP4L overexpression, we used high-titer lentiviral shRNA- and cDNA-expressing vectors prepared by Shanghai GenePharma Co (Shanghai, China). The shRNA sequence can be found in Supplementary Table 3. For lentivirus infection, cells were cultured overnight in media. The following day, 1 × 10^6^ cells were resuspended in 100 μl medium with lentivirus (multiplicity of infection (MOI)=100) and 5 μg ml^−1^ polybrene in 24-well culture plate. Infections were carried out for 6 h at 37 °C, 5% CO_2_. After the end of infection, 400 μl medium was added. Knockdown or overexpression was verified by western blotting after 4 and 2 days infection, respectively. For other genes transfer, cells were electroporated using an 4D-Nucleofector System (Lonza, Basel, Switzerland) according to the manufacturer's instructions. The transfection efficiency can reach up to 80% and verified by western blotting.

### Extracellular flux assays and ATP generation measurement

Oxygen consumption was assessed using MitoXpress-Xtra-HS (Cayman Chemical, Ann Arbor, MI, USA), a porphyrin-based phosphorescent oxygen-sensitive probe. Before assay, cells were transferred into fresh culture medium containing 1% FBS. Overall, 10 μl of probe was added and the cells were equilibrated at 37 °C. The assay was read using a Microplate Reader (Synergy 4 Hybrid, BioTek, Winooski, VT, USA). The maximal rate of oxygen consumption is proportional to the change in probe fluorescence during the linear phase of the assay. The lactic acid levels and ATP levels were determined by glycolysis cell-based assay kit (Cayman Chemical) and ATP Bioluminescence Assay Kit CLS II (Roche, Basel, Switzerland) according to the manufacturer's instructions.

### Mitochondrial and cytosolic ATP measurements

Mitochondrial and cytosolic ATP levels were measured using a luciferase probes PcDNA3-COX8-luc and PcDNA3lucLL/V, respectively. After 24 h of transfection, cells were rinsed in PBS, and resuspended in 90 μl of a 25 mM Tricine, 150 mM NaCl buffer, pH 7.4. Overall, 10 μl of 20 mM beetle luciferin (Promega, Madison, WI, USA; final concentration 2 mM) was added to the cell suspension and light emission measured in Microplate Reader (Synergy 4 Hybrid, BioTek). To normalize for the variability of luciferase expression in transfected cells, the relative luminescence values in each cell compartment were expressed as a ratio to the ‘total potential luminescence' measured on equal aliquots of the same lysed cells with a luciferase assay kit according to the manufacturer's protocol (Promega) in the presence of excess ATP (0.5 mM final concentration).

### Measurement of ROS levels

Endogenous ROS levels of normal T-cells and primary T-ALL cells were detected by labelling cells for 20 min at 37 °C with 10 μM redox-sensitive probe DCFH-DA (Life Technologies). After washing three times with PBS, cells were analyzed using flow cytometer (FACSAriaTM, BD).

### Quantitative real-time PCR and RT-PCR

Total RNA was isolated with TRIzol reagent according to the manufacturer's instructions. RNA samples were reverse transcribed using random hexamer primers in the presence of RNase inhibitor (Takara Bio). qRT-PCR was performed with SYBR Premex EX Taq (Takara Bio) using the 7,300 Sequence Detection System (Applied Biosystems, Foster City, CA, USA). A relative quantification analysis was performed using the ΔΔCt method, with actin as endogenous reference. Relative gene expression is presented as the ratio of target gene to reference. Primer sequences used were as follows: ORP4L (sense 5′- CCCTTCACTAAGGCCGCATC -3′, anti-sense 5′- GAACCCCAAGAGGAGTCTTCG -3′); actin (sense 5′- GGCATCCTCACCCTGAAGTA -3′, anti-sense 5′- AGGTGTGGTGCCAGATTTTC -3′). For RT-PCR, PCR primer sets were designed to amplify specific ORP4 isoforms: ORP4L, CGTTAAAGCCCCTGCCTCTTCTGC and GTGTTCATCACGCGGACAGCCTTG ; ORP4M, GAAGCGCCTTGGCATGAACCGTAG and GTGTTCATCACGCGGACAGCCTTG ; ORP4S, TGGTGTCCTGTGCCATTGTTAAAC and TTGGATGTGATGCGGAAGAGGG . PCR products were separated by 1.5% agarose gel electrophoresis and visualized with ethidium bromide.

### Analysis of the ORP4L-associated proteome

To obtain enough ORP4L antibody, rabbit antibody against human ORP4L were produced by immunizing New Zeal- and White rabbits with recombined protein carrying amino acid 382–485 of human ORP4L (NCBI Reference Sequence: NP_110385.1). Overall, 5 mg of this antibody and control IgG were coupled in CNBr activated sepharose. Total cell lysates from Jurkat T-cells stimulated with 10 μg ml^−1^ anti-CD3 for 5 min were immunoprecipitated with antibody-coupled sepharose. The ORP4L and its interacting proteins were eluted and subjected to SDS–PAGE followed by in-gel tryptic digestion and mass spectrometric identification as described previously^50^.

### Co-immunoprecipitation and immunodepletion

For co-immunoprecipitation assays, Jurkat T-cells were stimulated with 10 μg ml^−1^ of anti-CD3, and cell lysates were immunoprecipitated with antibodies as indicated. For immunodepletion assays, cell lysates were precipitated with anti-ORP4L antibody (Sigma-Aldrich), and supernatants were collected for co-immunoprecipitation with anti-Gα_q/11_.

### Immunofluorescence microscopy

Cells seeded onto coverslips were stimulated with anti-CD3 for the time indicated and fixed with 4% paraformaldehyde for 30 min at room temperature, followed by permeabilization with 0.1% Triton X-100 for 5 min, and blocked with 10% FBS for 30 min at room temperature. Cells were then incubated with primary antibodies in 5% FBS at 4 °C overnight. After washing three times (10 min each) with PBS, cells were incubated for 30 min with secondary antibody conjugated with fluorescence at 37 °C. The specimens were analyzed using a Zeiss (Oberkochen, Germany) LSM 510 Meta laser scanning confocal microscope system.

### Determination of phospholipase C activity

Phospholipase C activity was analyzed by using the EnzChekR Direct Phospholipase C Assay Kit (Molecular Probes, Eugene, OR, USA) according to the manufacturer's instructions.

### Plasma membrane preparations

Cell pellets were thawed and resuspended in 20 volumes of 20 mM sodium phosphate buffer, pH 7.4, and were homogenized twice for 30 s at high speed using a Tissuemizer. Homogenates were centrifuged at 200*g* for 15 min at 4 °C. The supernatant was removed and reserved on ice. This procedure was repeated twice and the pooled supernatants were then centrifuged at 40,000*g* for 45 min at 4 °C. Membranes were suspended at 5 mg protein ml^−1^ and were stored at −80 °C.

### Anti-CD3 mediated [^35^S]GTPγS binding assay

Membrane proteins (40 μg) were diluted in a final volume of 250 μl assay buffer composed of 50 mM Tris pH 7.4, 100 mM NaCl, 0.2 mM EGTA, 3 mM MgCl_2_, 1 mM DTT, 50 μM GDP, 0.5 nM [^35^S]GTPγS and 10 μg ml^−1^ of anti-CD3 antibody. Following an incubation period of 120 min at a temperature of 25 °C while shaking at 1,000 r.p.m., the reaction was terminated by rapid filtration through Whatman GF/B filters, followed by six washes with 1 ml of ice-cold washing buffer (50 mM Tris pH 7.4, 100 mM NaCl, 0.2 mM EGTA, 3 mM MgCl_2_). Subsequently, filters were placed in scintillation vials containing 5 ml liquid scintillation cocktail and decays per minute of the filter-bound [^35^S]GTPγS were measured by a Liquid Scintillation Analyzer (TriCarb B2810TR, Perkin Elmer).

### Measurement of IP_3_ production

IP_3_ was measured using the HitHunter IP_3_ Fluorescence Polarization Assay Kits (DiscoverRx Tech, Fremont, CA, USA). Briefly, 2 × 10^4^ cells in black 384-well plates (Greiner, Germany) were treated with anti-CD3 for the designated times, and the cellular reaction was terminated by adding 0.2 N perchloric acid. The plate was shaken at 650 r.p.m. for 15 min. The IP_3_ tracer was subsequently added to each well, and the IP_3_ binding protein was added to the plate. The polarized fluorescence from the IP_3_ tracer was read on a Microplate Reader (Synergy 4 Hybrid, BioTek, ). The IP_3_ concentration was calculated from the IP_3_ standard curve.

### Calcium fluorometry

Cells (1 × 10^6^ cells ml^−1^) were incubated with 1 μM Fura-2-AM for 30 min at 37 °C in extracellular calcium buffer (ECB, 130 mM NaCl, 5 mM KCl, 1.5 mM CaCl_2_, 1 mM MgCl_2_, 25 mM Hepes, pH 7.5, 1 mg ml^−1^ BSA, and 5 mM glucose) in dark, after which they were collected and resuspended in ECB for an additional incubation at 25 °C for 30 min to permit dye de-esterification. Cells were then collected and resuspended to 2 × 10^6^ cells ml^−1^ in ECB. Cells in 100 μl ECB were transferred to a 96-well black-wall cell culture plates and fluorescence was continuously recorded at 25 °C (alternating 340 and 380 nm excitation, 510 nm emission) in a Microplate Reader (Synergy 4 Hybrid, BioTek).

### Ca^2+^ imaging

Cells (0.5 × 10^6^ cells ml^−1^) were plated onto a glass-bottomed dishes and incubated with 1 μM Fluo-4-AM for 30 min at 37 °C for [Ca^2+^]_c_ or 2 μM Rhod-2-AM for 60 min at 37 °C for [Ca^2+^]_m_ in ECB. The buffer was replaced and incubation continued for 20 min at 37 °C to permit dye de-esterification. Culture dishes were mounted on the stage of an inverted confocal microscope (Zeiss LSM 510 Meta) equipped with a 40 × oil objective. Calcium measurements were performed in fresh RPMI 1640 complete medium with 10% FBS without phenol red at 37 °C with 5% CO_2_. Cells were excited with low-intensity 488-nm (for Fluo-4-AM) or 546-nm (for Rhod-2-AM) laser excitation and images were acquired at 2-s intervals under time-lapse mode. Spontaneous cytosolic calcium oscillations were recorded for 20 min. Image data were subsequently analyzed using ImageJ (National Institutes of Health) and were presented as a ratio of F/F0 in the final results, where F0 represents baseline fluorescence intensity in each cell.

### Measurements of Ca^2+^ influx and ER Ca^2+^ content

For Ca^2+^ influx experiments, ECB containing 10 mM EGTA instead of Ca^2+^ was used. Cells (1 × 10^6^ cells ml^−1^) were incubated with 1 μM Fura-2-AM for 30 min at 37 °C in ECB in dark, after which they were collected and resuspended in ECB for an additional incubation at 25 °C for 30 min to permit dye de-esterification. Cells were stimulated with 10 μg ml^−1^ anti-CD3 antibody, then the medium was replaced by ECB containing 2 mM CaCl_2_ to measure Ca^2+^ influx. For ER Ca^2+^ recording, we used the low-affinity Ca^2+^ indicator Fluo-5N-AM. Following the incubation with 10 μM Fluo-5N-AM for 2 h and de-esterified for 1 h at 37 °C, cells were permeabilized by saponin (50 μg ml^−1^) for 20 s. Cells were plated onto a glass-bottomed dishes and excited with low-intensity 488-nm laser excitation and images were acquired at 2-s intervals under time-lapse mode in confocal microscope (Zeiss LSM 510 Meta) equipped with a 40 × oil objective. Image data were subsequently analyzed using ImageJ (National Institutes of Health) and were presented as a ratio of F/F0 in the final results, where F0 represents baseline fluorescence intensity in each cell. Total Ca^2+^ concentration was measured in three regions of the cell, the cytosolic, the mitochondrial and the ER. Ca^2+^ concentration was calculated based on the *K*_d_ for Fura-2 of 220 nM, Rhod-2 of 570 nM and Fluo-5N of 90 μM.

### Mitochondrial membrane potential measurement and NADH imaging

Inner mitochondrial membrane potential (Δψ) was assessed with the potential sensitive cationic fluorescent dye JC-1 (Sigma-Aldrich). Overall, 1/10 volume of 20 μM JC-1 was added to cultured cells followed by incubation at 37 °C with 5% CO_2_ for 15 min. Cells were washed and resuspended to 2 × 10^6^ cells ml^−1^ in ECB, and cellular fluorescence was measured in a Microplate Reader (Synergy 4 Hybrid, BioTek) set at an excitation/emission wavelengths of 525/590 nm (JC-1 aggregates) and 490/530 nm (JC-1 monomer). An aliquot of cells for each sample without staining with JC-1 was measured to determine background fluorescence. JC-1 fluorescence was also visualized using fluorescence microscopy (Nikon Eclipse TE2000-s). Stained cells were collected and resuspended in 10 μl complete medium, mounted on a coverslip, and images were acquired from three or more randomly chosen fields with G-2 A cube (Ex: 510–560 nm, Em: >590 nm) for JC-1 aggregates (red) first and then with FITC cube (Ex: 465–495 nm, Em: 515–555 nm) for JC-1 monomer (green). Images from aliquots of cells stained without JC-1 were acquired as background signal. Data are expressed as ratio of red/green after subtracting the background.

NAD(P)H autofluorescence was imaged by fluorescence microscopy. All subsequent incubation and wash steps were carried out in ECB. Cells were washed, suspended to 0.5 × 10^6^ cells ml^−1^, and 0.25 ml of suspended cells were applied on coverslip dishes (25-mm) for 10 min at room temperature and images were acquired with DAPI cube (Ex: 340–380 nm, Em: 435–485 nm) with 40 × objective for fluorescence microscopy (Nikon Eclipse TE2000-s). Pictures were taken with the same excitation intensity and exposure time. NAD(P)H fluorescence intensity per cell was analyzed with ImageJ (National Institutes of Health).

### Cell proliferation and cell death assay

For cell proliferation assay, 5,000 Jurkat T-cells infected with control or shORP4L lentivirus for 24 h were plated in 96-well flat bottom plates with RPMI 1640 medium supplemented with 10% of FBS. After 1–4 days' culture, cell numbers were evaluated by Cell Counting kit-8 (WST-8 assay, Dojindo, Molecular Technologies, Rockville, MD, USA) following the manufacturer's protocol. Cell number was calculated by standard curve method, and the averages of at least three times independent experiments were presented. Cell death was analyzed by LIVE/DEAD Fixable Dead Cell Stain Kits (Life Technology) according to the manufacturer's instructions. Briefly, cells were washed once with PBS, and then incubated with LIVE/DEAD Fixable Dead Cell Stain in PBS for 30 min at room temperature in the dark. After washing with PBS with 1% FBS, cells were resuspended in PBS with 1% FBS and analyzed using flow cytometer (FACSAriaTM, BD).

### *In vivo* animal studies

This study has been conducted in accordance with the ethical standards and according to the Declaration of Helsinki and according to national and international guidelines and has been approved by the institutional ethics committee of Jinan University. Mice were kept under pathogen-free conditions in the Laboratory Animal Center, Sun Yat-sen University. To establish an engraftment model in female NOD/SCID mice, 2 × 10^7^ primary T-ALL cells were infected by shNT and shORP4L lentivirus. After 72 h infection, cells were resuspended in 200 μl PBS and injected via the tail vein of 6-weeks age female animals, randomly. At 2 weeks post-injection, mice were sacrificed, and engraftment was assayed by flow cytometric detection of human CD45^+^ T-ALL cells.

### Detection of ER–mitochondrial contact sites

For transmission electron microscopy, cells were fixed with 2.5% glutaraldehyde in 0.1 M sodium cacodylate buffer for 1–2 h, contrasted with 1% osmium tetroxide, dehydrated through a graded ethanol series, and embedded in plastic. Ultrathin sections were examined with a FEI Tecnai 10 (Hillsboro, OR, USA) transmission electron microscope. For ultra-high resolution fluorescence microscopy, cells were transfected with DsRed-Mito and GFP-ER for 24 h before fixation. Fluorescence images were acquired using DeltaVision Elite system (GE Healthcare, Little Chalfont, Buckinghamshire, UK). Co-localization between the ER and mitochondrial markers was quantified by calculating Pearson's correlation coefficient.

### BODIPY-cholesterol labelling and chase

BODIPY-cholesterol was complexed with methyl-β-cyclodextrin at a molar ratio of sterol/cyclodextrin 1/10, probe sonicated for 3 × 15 min on ice and centrifuged at 10,000*g* for 2 × 30 min. Jurkat T-cells were transfected with DsRed-Mito or DsRed-ER plasmid and cultured for 24 h before BODIPY-chol labelling. Cells were seeded onto coverslips and incubated in serum-free medium at 37 °C. Then 1 ul MβCD/BODIPY-chol complex was added as in 1:2,000 dilutions, resulting in a final concentration of 0.185 mM MβCD and ∼1 μM BODIPY-chol. After labelling for 10 min at 37 °C, the cells were washed three times with PBS. The cells were chased and imaged in a CO_2_-independent medium at 37 °C. Images were taken with LSM 510 Meta laser scanning confocal microscope system. Co-localization of BODIPY-chol and the red organelles marker, as expressed by the Pearson's correlation coefficient was used as a measure of the relative amount of BODIPY-chol in the organelle.

### Western blot analysis

Cellular total protein samples were mixed with loading sample buffer, boiled for 10 min, and subjected to SDS–PAGE followed by transfer onto PVDF membranes (Millipore, Life Science). After blocking and incubations of the membranes with primary antibodies and HRP-secondary antibody conjugates (Bio-Rad Laboratories), the blots were developed by enhanced chemiluminescence (Millipore, Life Science). Proteins were quantified by densitometry using ImageJ (National Institutes of Health) and the data normalized using the β-actin signal. The original uncropped scans of all the western blot results are provided in Supplementary Fig. 13.

### Statistics

The data are expressed as mean±s.d. All comparisons between groups were made by unpaired two-tailed Student's *t* test. *P* values of <0.05 were considered statistically significant.

### Data availability

The authors declare that all data supporting the findings of this study are available within the article and its Supplementary Materials, or from the corresponding authors upon request.

## Additional information

**How to cite this article:** Zhong, W. *et al*. ORP4L is essential for T-cell acute lymphoblastic leukemia cell survival. *Nat. Commun.* 7:12702 doi: 10.1038/ncomms12702 (2016).

## Supplementary Material

Supplementary Information Supplementary Figures 1-13 and Supplementary Tables 1-4.
