# Supplementary material for: ORP4L is essential for T-cell acute lymphoblastic leukemia cell survival
Source: Nat Commun. 2016 Sep 1;7:12702. doi: 10.1038/ncomms12702 (PMC5025801; doi:10.1038/ncomms12702)
Supplement: Supplementary Information — Supplementary Figures 1-13 and Supplementary Tables 1-4. [file ncomms12702-s1.pdf]

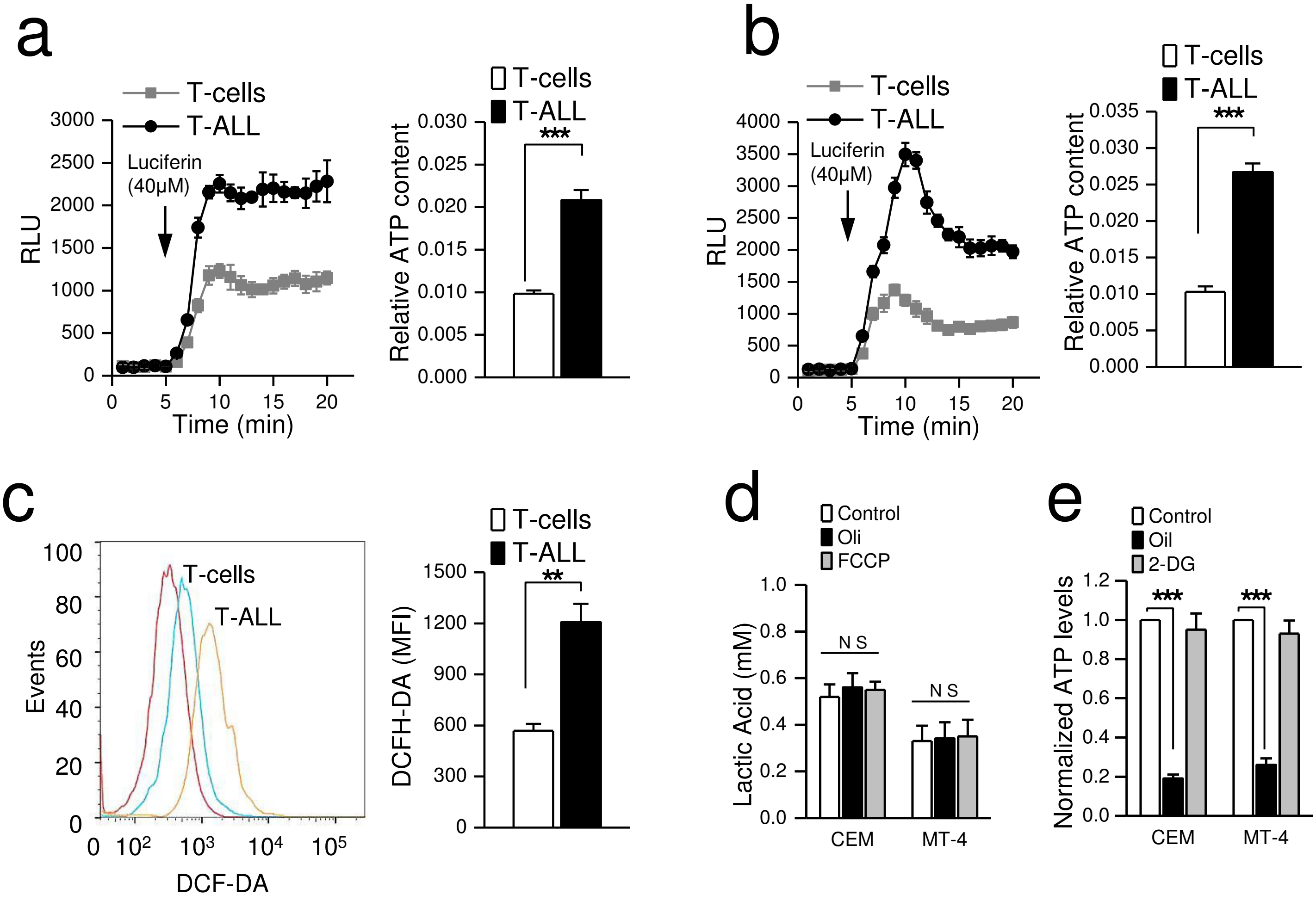

Supplementary Figure 1. Increased mitochondrial metabolism in T-ALL cells. (a, b), mitochondrial (a) and cytosolic (b) ATP were measured in normal T-cells and primary T-ALL cells transfected with mitochondrial luciferase (PcDNA3-COX8-luc) or cytosolic (PcDNA3lucLL/V) luciferase. Representative luciferase luminescence curves as a function of time and quantification of the luminescence as a measure of ATP content in normal T-cells (n=3) and primary T-ALL cells (n=4) are shown. **(c)** A representative histogram of a FACS-analysis showing normal T-cells and primary T-ALL cells stained with a ROS-sensitive dye (DCFH-DA) and an unstained control. An overview of the DCFH-DA fluorescence intensity (MFI) in normal T-cells (n=3) and primary T-ALL cells (n=4) are shown. **(d)** Lactic acid levels in CEM and MT-4 cells after treatment with the oxidative phosphorylation inhibitor oligomycin (Oli, 5 μM) or FCCP (5 μM) for 4 h. **(e)** ATP levels were measured in CEM and MT-4 cells after treatment with oligomycin (Oli, 5 μM) or the glycolysis inhibitor 2-DG, (5 μM) for 6 h. The data represent mean ± S.D. value from an experiment performed in triplicate. $**p < 0.01$, $***p < 0.001$, NS, not significant, Student's t test.

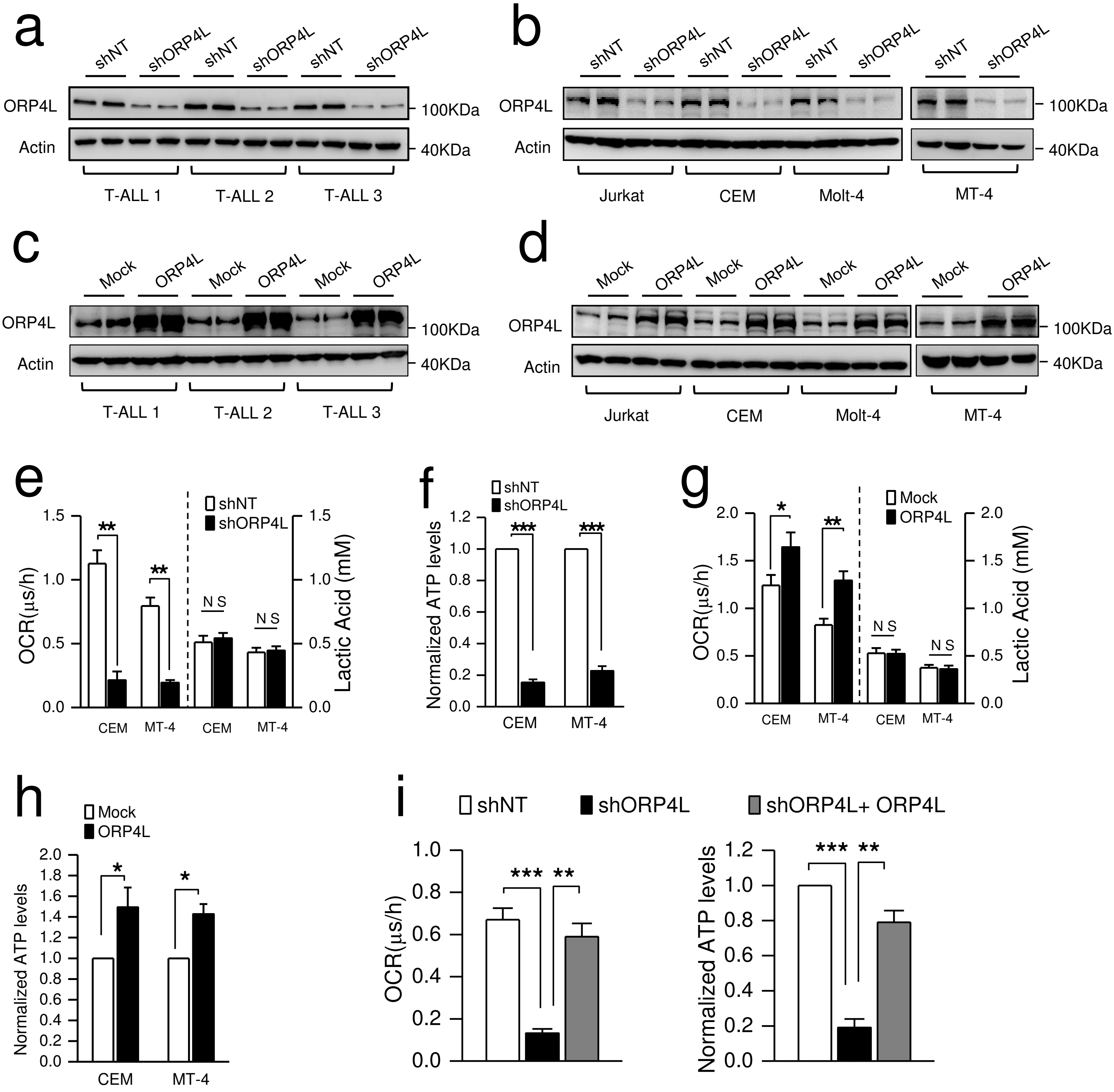

Supplementary Figure 2. Western blot analysis of ORP4L expression and the role of ORP4L in mitochondrial metabolism of T-ALL cells. (a, b) Western blot analysis of ORP4L knockdown efficiency in primary T-ALL cells (a) and T-ALL cell lines (b). **(c, d)** Western blot analysis of ORP4L overexpression in primary T-ALL cells (c) and T-ALL cell lines (d). **(e)** Baseline OCR and lactic acid levels in CEM and MT-4 cells with ORP4L knockdown. **(f)** ATP levels in CEM and MT-4 cells with ORP4L knockdown. **(g)** Baseline OCR and lactic acid levels in CEM and MT-4 cells with ORP4L overexpression. **(h)** ATP levels in CEM and MT-4 cells with ORP4L overexpression. **(i)** Baseline OCR (left) and ATP levels (right) in shNT or shORP4L lentivirus-infected Jurkat T-cells and shORP4L-infected cells re-transfected with ORP4L. The data represent mean ± S.D. from an experiment performed in triplicate. *$p < 0.05$, **$p < 0.01$, ***$p < 0.001$, NS, not significant, Student's t test.

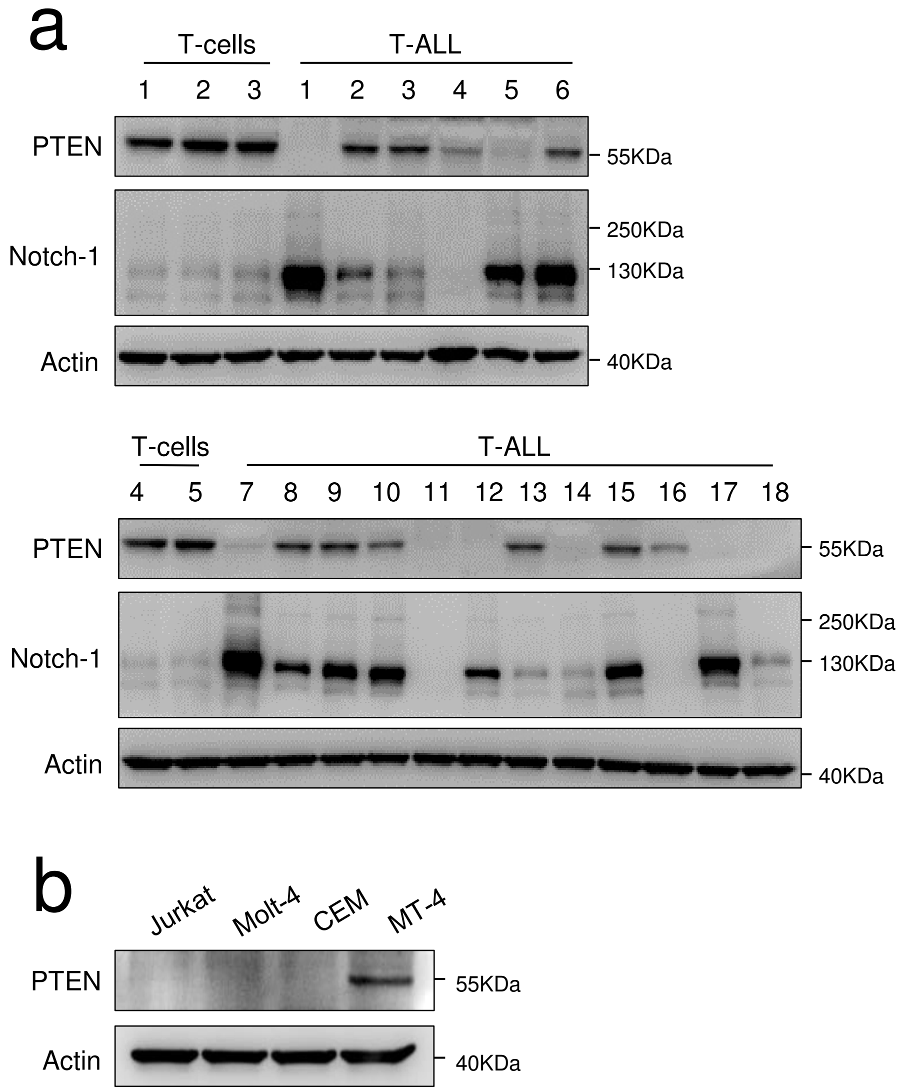

Supplementary Figure 3. Western blot analysis of PTEN and Notch-1 status in normal T-cells and primary T-ALL cells (a), and of PTEN status in the T-ALL cell lines used (b).

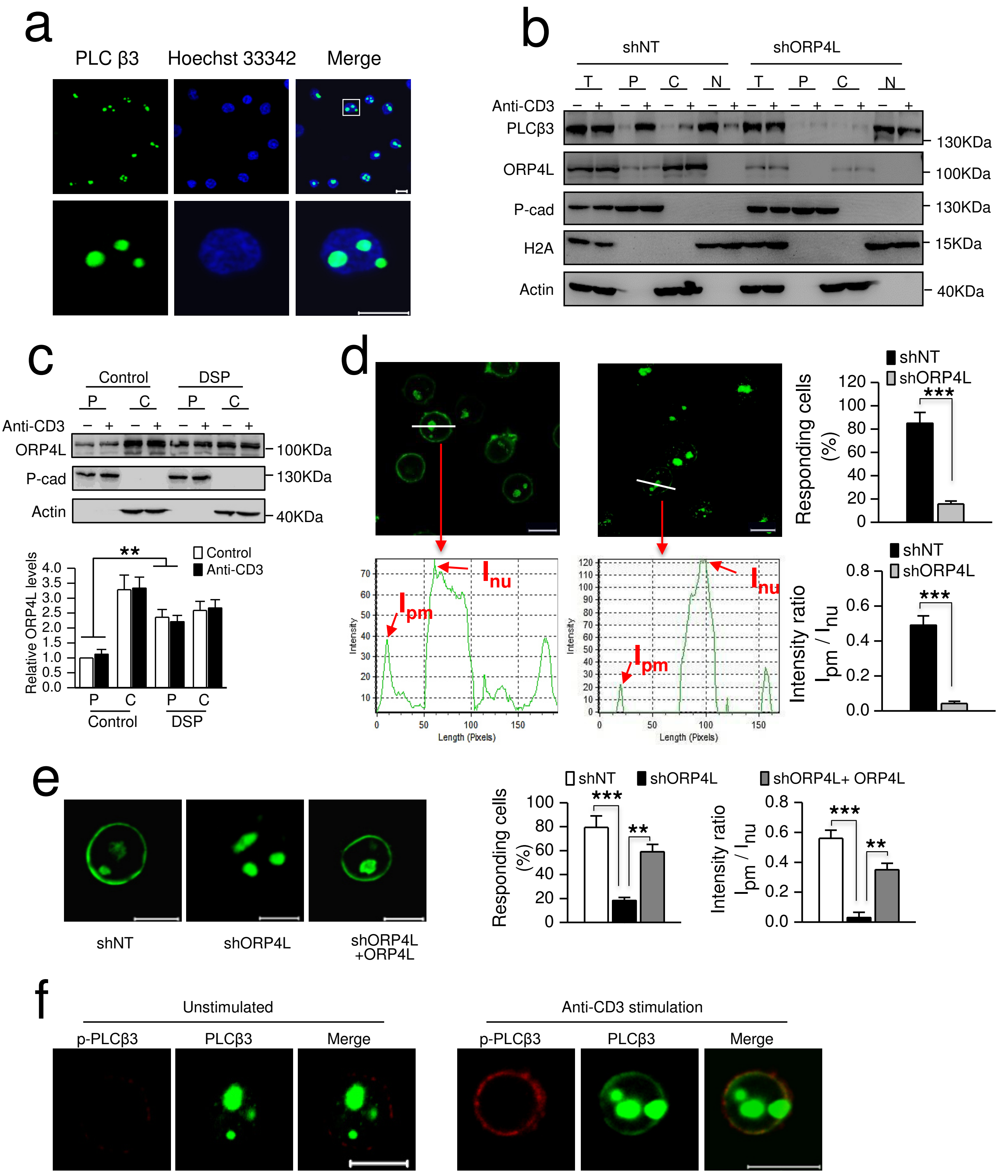

a
PLC β3
Hoechst 33342
Merge
b
shNT
shORP4L
T
P
C
N
Anti-CD3
PLCβ3
ORP4L
P-cad
H2A
Actin
130KDa
100KDa
130KDa
15KDa
40KDa
c
Control
DSP
P
C
Anti-CD3
ORP4L
P-cad
Actin
100KDa
130KDa
40KDa
Relative ORP4L levels
Control
Anti-CD3
**
d
Intensity
Length (Pixels)
Inu
Ipm
shNT
shORP4L
Responding cells (%)
***
Intensity ratio Ipm / Inu
e
shNT
shORP4L
shORP4L +ORP4L
shNT
shORP4L
shORP4L+ ORP4L
Responding cells (%)
Intensity ratio Ipm / Inu
***
**
f
Unstimulated
Anti-CD3 stimulation
p-PLCβ3
PLCβ3
Merge

Supplementary Figure 4. ORP4L knockdown prevents PLCβ3 translocation to the plasma membrane and activation. (a) Immunofluorescence staining with anti-PLCβ3 showing nuclear localization of the protein. Hoechst33342 staining indicates the nucleus. Scale bars, 10 μm. **(b)** Control and ORP4L knockdown Jurkat T-cells were treated for 5 min in the presence or absence of 10 μg/mL anti-CD3. The total (T), plasma membrane (P), cytosolic (C) and nuclear (N) fractions were analyzed by western blot. Pan-cadherin, histone H2A and actin were used as loading controls for the plasma membrane, nuclear and cytosolic fractions, respectively. **(c)** Jurkat T-cells were treated for 5 min in the presence or absence of 10 μg/mL anti-CD3, and then cells were cross-linked by 1mM DSP for 30 min before lysis. The plasma membrane (P) and cytosolic (C) fractions were analyzed by western blot. **(d)** PLCβ3 translocation in control and ORP4L knockdown Jurkat T-cells upon 10 μg/mL anti-CD3 stimulation for 5 min (upper, left). The percentage of cells responding to anti-CD3 is represented as a histogram (upper, right) (100 cells from three experiments with 10 random fields/experiment). A line intensity profile across the cell was obtained in a given image (upper, left). Representative intensity profiles are shown (lower, left). The relative fluorescence intensity ratio of plasma-membrane-adjacent area (I_{pm}) versus nuclear area (I_{nu}) are shown (lower, right; 50 cells from three experiments with 10 random fields/experiment). **(e)** PLCβ3 translocation in shNT or shORP4L lentivirus-infected Jurkat T-cells and shORP4L -infected cells re-transfected with ORP4L. Cells were stimulated for 5 min in the presence or absence of 10 μg/mL anti-CD3. The percentage of cells responding to anti-CD3 (100 cells from three experiments with 10 random fields/experiment) and the relative fluorescence intensity ratio of plasma-membrane-adjacent area (I_{pm}) versus nuclear area (I_{nu}) are shown (50 cells from three experiments with 10 random fields/experiment). Scale bars, 10 μm. **(f)** Confocal microscopy analysis of PLCβ3 (green) and p-PLCβ3 (red) localization in Jurkat T-cells. Cells were stimulated for 5 min in the presence or absence of 10 μg/mL anti-CD3. Scale bars, 10 μm. The data represent mean ± S.D. from an experiment performed in triplicate. $^{**}p < 0.01$, $^{***}p < 0.001$, Student's t test.

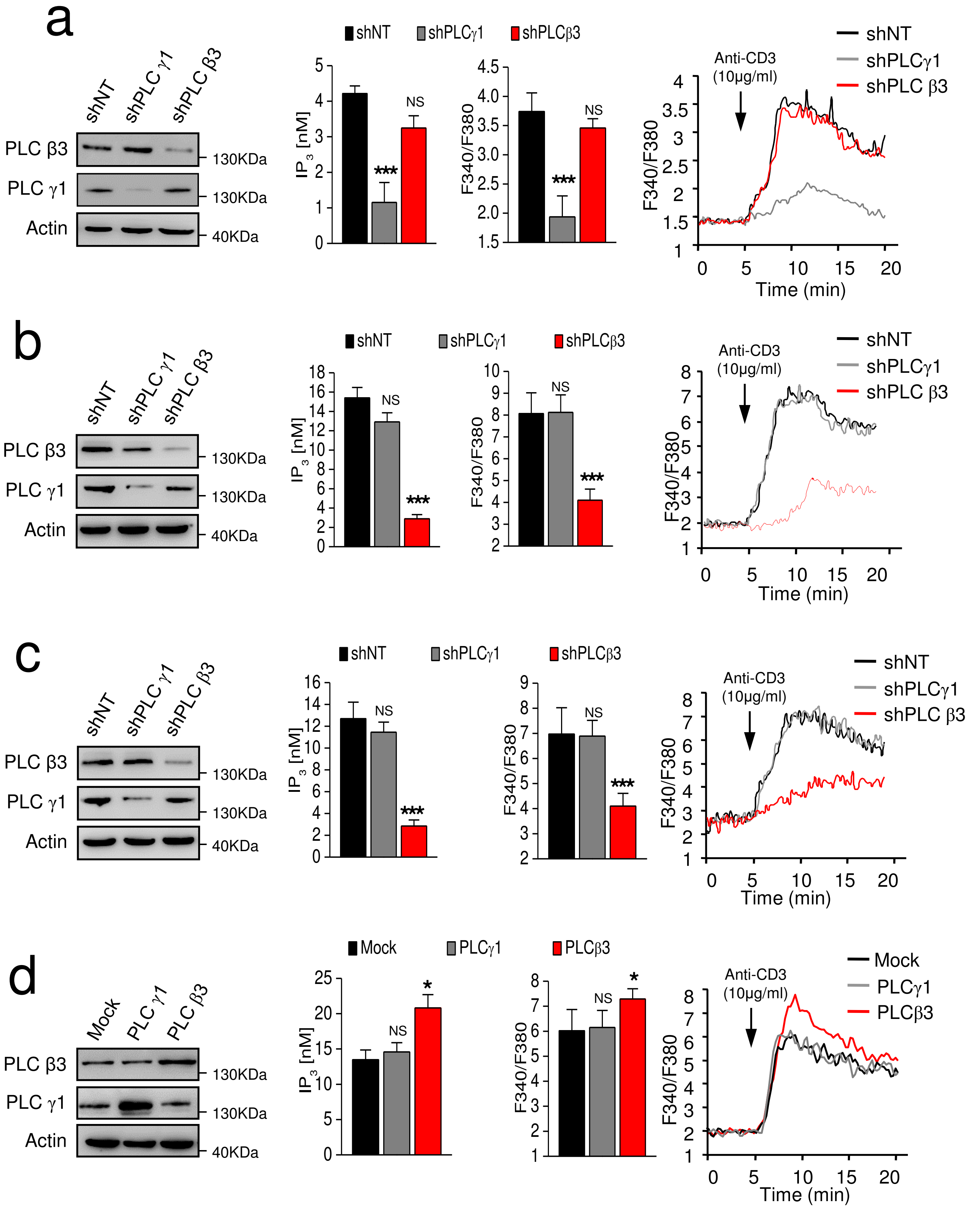
a
shNT
shPLC γ1
shPLC β3
PLC β3
PLC γ1
Actin
130KDa
130KDa
40KDa
shNT
shPLCγ1
shPLCβ3
IP3 [nM]
***
NS
F340/F380
***
NS
Anti-CD3
(10μg/ml)
shNT
shPLCγ1
shPLC β3
F340/F380
Time (min)
b
shNT
shPLC γ1
shPLC β3
PLC β3
PLC γ1
Actin
130KDa
130KDa
40KDa
shNT
shPLCγ1
shPLCβ3
IP3 [nM]
NS
***
F340/F380
NS
***
Anti-CD3
(10μg/ml)
shNT
shPLCγ1
shPLC β3
F340/F380
Time (min)
c
shNT
shPLC γ1
shPLC β3
PLC β3
PLC γ1
Actin
130KDa
130KDa
40KDa
shNT
shPLCγ1
shPLCβ3
IP3 [nM]
NS
***
F340/F380
NS
***
Anti-CD3
(10μg/ml)
shNT
shPLCγ1
shPLC β3
F340/F380
Time (min)
d
Mock
PLC γ1
PLC β3
PLC β3
PLC γ1
Actin
130KDa
130KDa
40KDa
Mock
PLCγ1
PLCβ3
IP3 [nM]
NS
*
F340/F380
NS
*
Anti-CD3
(10μg/ml)
Mock
PLCγ1
PLCβ3
F340/F380
Time (min)

Supplementary Figure 5. PLCβ3 is essential for Ca^{2+} release in T-ALL cells. Measurement of anti-CD3 induced IP_3 production and $[Ca^{2+}]_i$ transients upon PLCβ3 or PLCγ1 knockdown in (a) normal T-cells, (b) primary T-ALL cells, and (c) Jurkat T-cells. **(d)** Measurement of anti-CD3 induced IP_3 production and $[Ca^{2+}]_i$ transients upon PLCβ3 or PLCγ1 overexpression in Jurkat T-cells. IP_3 concentration was determined by stimulation of cells with 10 μg/mL anti-CD3 for 5 min. $[Ca^{2+}]_i$ were recorded as F340/380 ratio using Fura2-AM in cells with 10 μg/mL of anti-CD3 stimulation. Average $[Ca^{2+}]_i$ responses and quantification of $[Ca^{2+}]_i$ peak amplitudes of normal T-cells or primary T-ALL cells are shown. For the Jurkat T-cells the data represent mean ± S.D. value from an experiment performed in triplicate, and for primary T-cells and T-ALL cells mean ± S.D. value of n=3 primary T-ALL specimens. *$p< 0.05$, ***$p < 0.001$, NS, not significant, Student's t test.

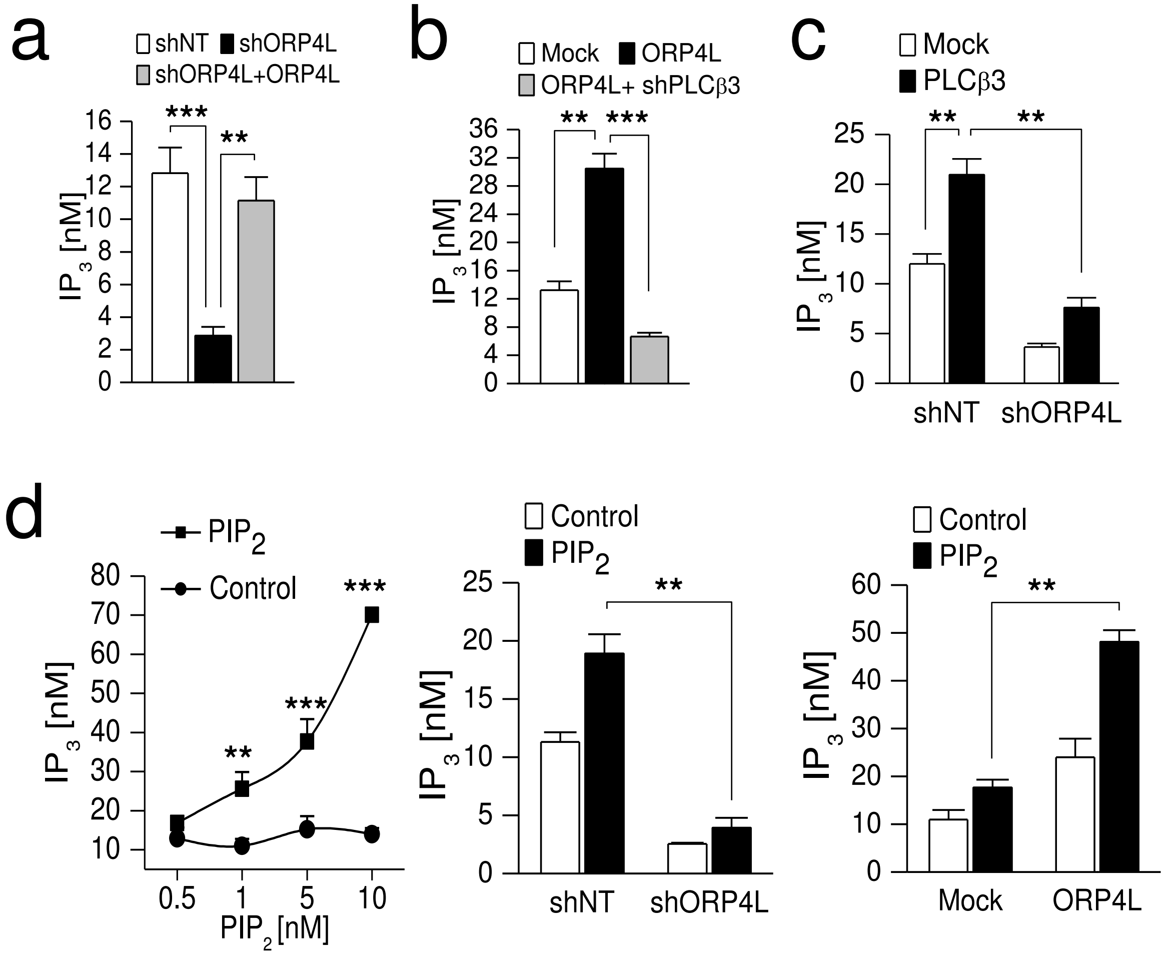

Supplementary Figure 6. ORP4L modulates IP_3 production. (a) IP_3 production in shNT or shORP4L transduced Jurkat T-cells and shORP4L transduced cells re-transfected with ORP4L. **(b)** IP_3 production in Jurkat T-cells overexpressing ORP4L alone or in combination with shPLCβ3. **(c)** IP_3 production in shNT or shORP4L transduced Jurkat T-cells with or without PLCβ3 overexpression. **(d)** IP_3 production in wild-type Jurkat T-cells incubated with increasing concentrations of exogenous PIP_2 (left), and ORP4L knockdown (middle) or ORP4L overexpressing (right) cells after treatment with 1 nM PIP_2. Cells were incubated with PIP_2 for 1 h before 5 min stimulation with 10 μg/mL anti-CD3. The data represent mean ± S.D. value from an experiment performed in triplicate. $**p < 0.01$, $***p < 0.001$, Student's t test.

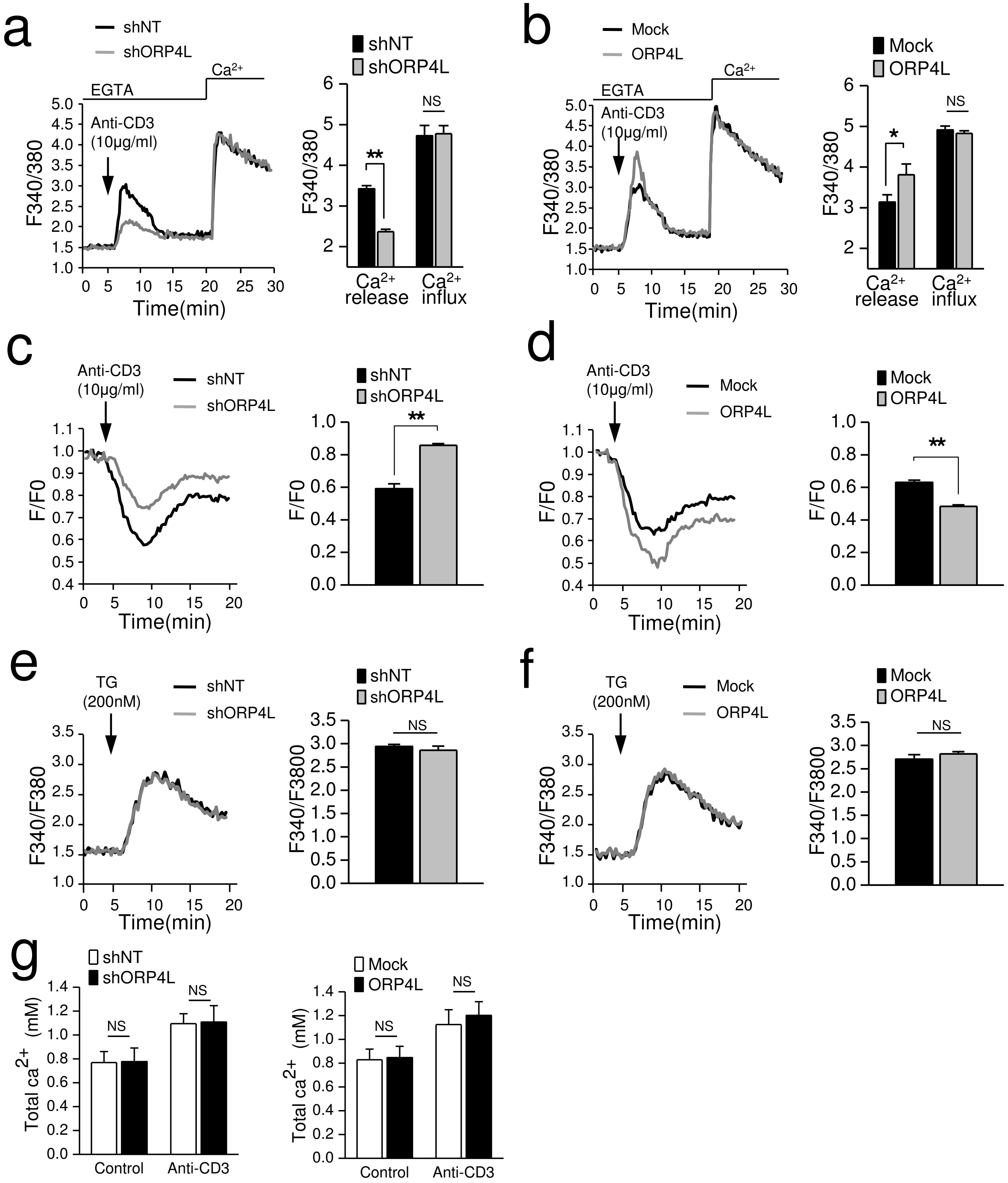
a
shNT
shORP4L
EGTA
Ca2+
Anti-CD3
(10μg/ml)
F340/380
Time(min)
Ca2+ release
Ca2+ influx
NS
**
b
Mock
ORP4L
*
c
F/F0
d
e
TG
(200nM)
F340/F380
F340/F3800
f
g
Total ca2+ (mM)
Control
Anti-CD3

Supplementary Figure 7. ORP4L regulates Ca^{2+} release from ER in Jurkat T-cells. (a, b) Jurkat T-cells subjected to ORP4L knockdown (a) or overexpression (b) were stimulated with 10 μg/mL anti-CD3 in ECB (containing10 mM EGTA) without Ca^{2+}. Changes in $[Ca^{2+}]_i$ were recorded as the F340/380 ratio using Fura2-AM. After stimulation, ECB containing 2 mM $CaCl_2$ instead of EGTA were used to analyze Ca^{2+} influx. Average $[Ca^{2+}]_i$ responses and quantification of $[Ca^{2+}]_i$ peak amplitudes are shown. **(c, d)** Jurkat T-cells subjected to ORP4L knockdown (c) or overexpression (d) were stimulated with 10 μg/mL anti-CD3 in ECB. Changes in $[Ca^{2+}]_i$ were recorded as the F/F0 ratio using Fluo5N-AM. Average $[Ca^{2+}]_E$ responses and quantification of $[Ca^{2+}]_E$ peak amplitudes are shown. **(e, f)** Jurkat T-cells subjected to ORP4L knockdown (e) or overexpression (f) were stimulated 100 nM TG in ECB. Changes in $[Ca^{2+}]_i$ were recorded as the F340/380 ratio using Fura2-AM. **(g)** Total amount of Ca^{2+} in Jurkat T-cells subjected to ORP4L knockdown and overexpression in the presence or absence of 10 μg/mL anti-CD3 stimulation. The data represent mean ± S.D. value from an experiment performed in triplicate. *$p < 0.05$, **$p < 0.01$, NS, not significant, Student's t test.

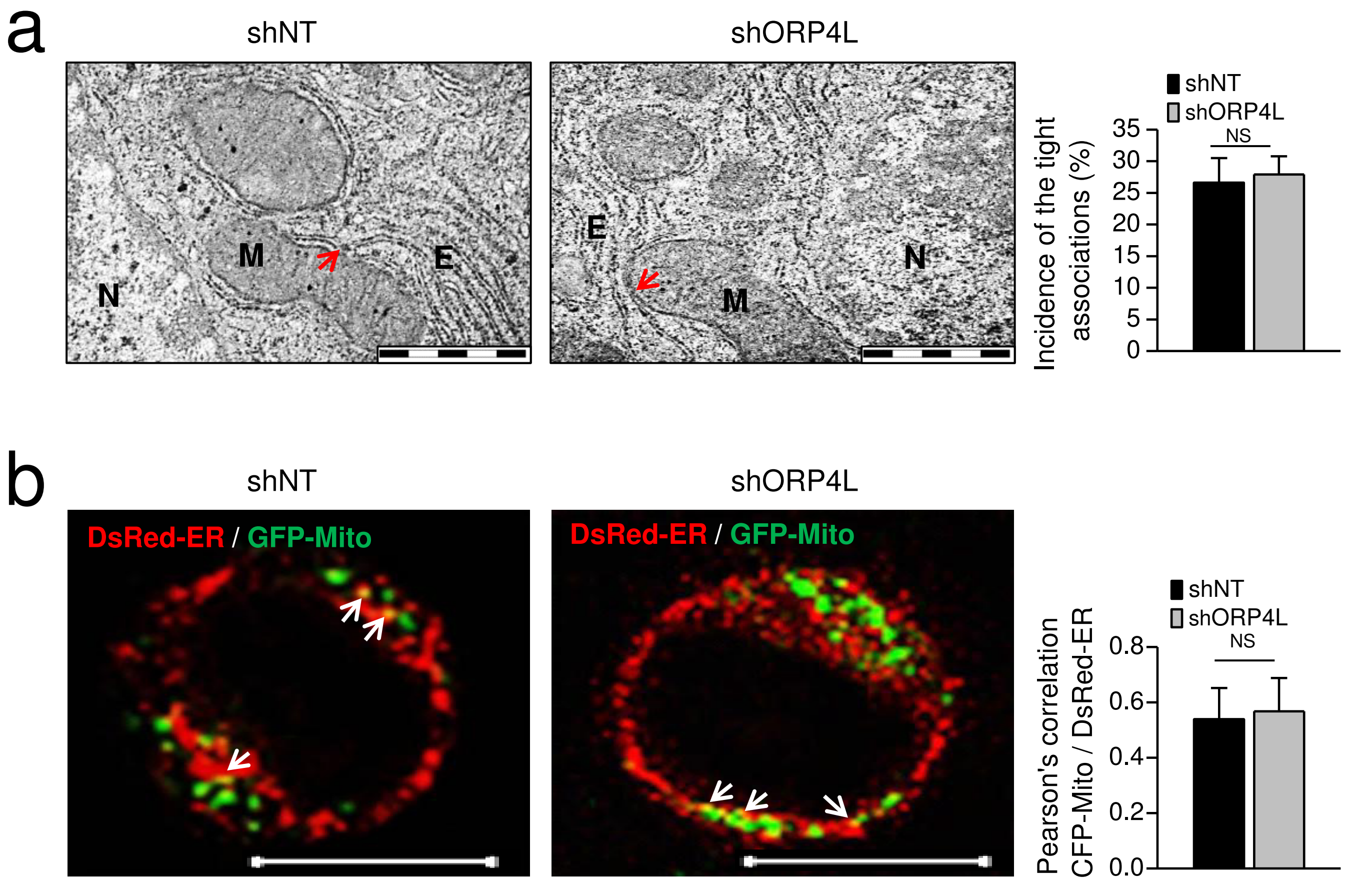

Supplementary Figure 8. ORP4L knockdown failed to change the quantity of ER-mitochondrial contacts in Jurkat T-cells. (a) TEM imaging of ER-mitochondrial contact sites (red arrowheads depicting the close contacts) in control and ORP4L knockdown Jurkat T-cells, and measurements of the ER–mitochondria interface (N, nucleus; M, mitochondrion; E, endoplasmic reticulum), Scale bars, 200 nm. **(b)** Confocal microscopy analysis of the colocalization of ER (red) and mitochondria (green). Scale bars, 10 µm. Pearson's correlation of the ER and mitochondrial signals is represented (35–40 cells were analyzed per condition). The data represent mean ± S.D. value from an experiment performed in triplicate. NS, not significant, Student's t test.

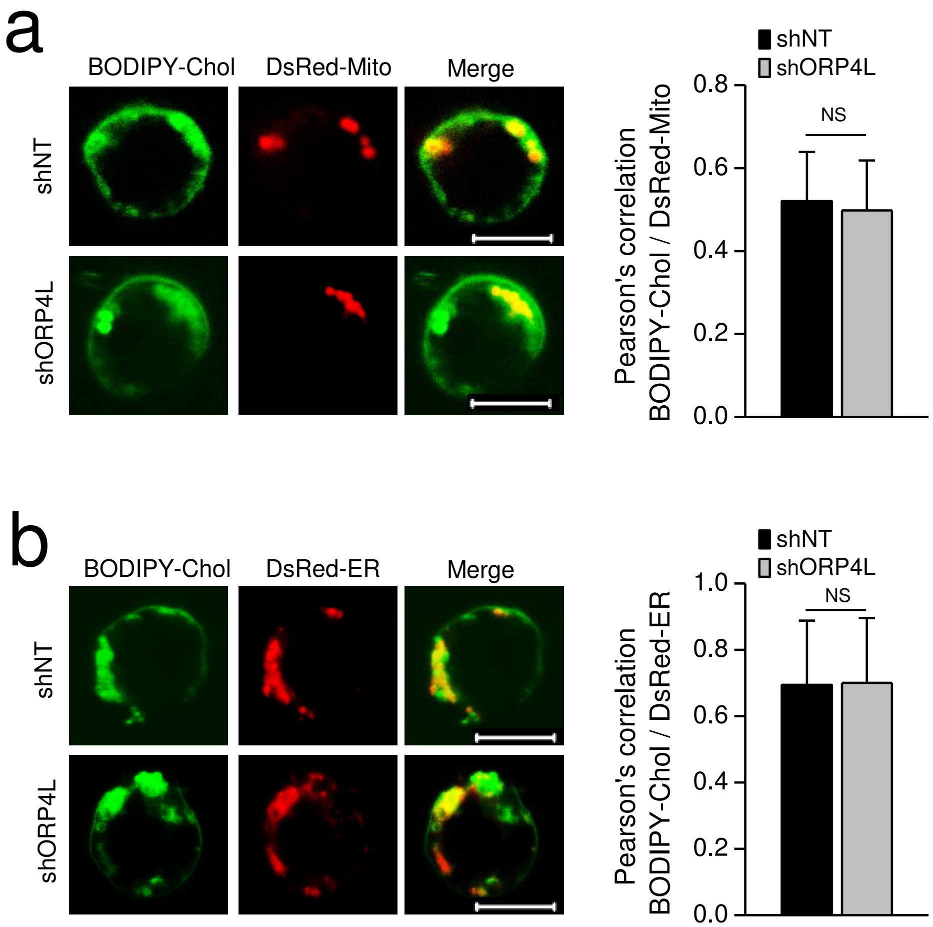

Supplementary Figure 9. ORP4L knockdown does not change the transport of BODIPY-cholesterol from the PM to ER or mitochondria. Control and ORP4L knockdown Jurkat T-cells were transfected with mitochondrial (DsRed-Mito) (a) or ER (DsRed-ER) marker (b) for 24 h. The cells were then labeled for 10 min with BODIPY-chol, chased for 60 min, and confocal images were taken after the chase. Scale bars, 10 µm. Bars indicate Pearson's correlation of BODIPY-chol and each organelle DsRed marker (35–40 cells were analyzed per condition). The data represent mean ± S.D. value from an experiment performed in triplicate. NS, not significant, Student's t test.

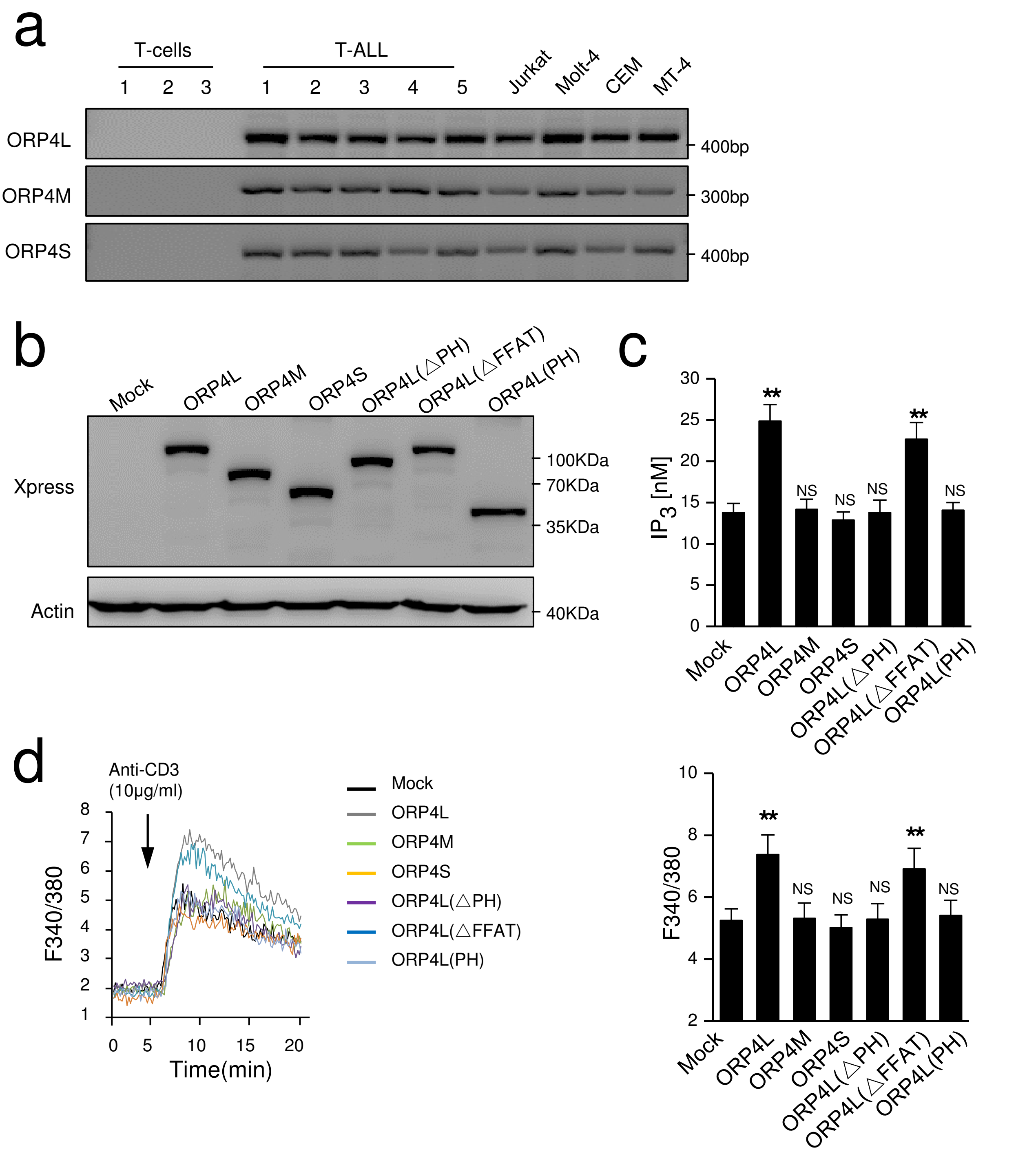

Supplementary Figure 10. The effects of ORP4M, ORP4S and truncated or mutated ORP4L constructs on Ca^{2+} signaling. (a) RT-PCR analysis of ORP4L, ORP4M and ORP4S expression in normal T-cells, primary T-ALL cells and T-ALL cell lines. **(b)** Western blot verifying overexpression of the constructs used in Jurkat T-cells, as detected by Xpress epitope tag antibody. **(c, d)** IP_3 production (c) and Ca^{2+} response (d) upon anti-CD3 stimulation in Jurkat T-cells overexpressing the indicated constructs. The data represent mean ± S.D. value from an experiment performed in triplicate. $^{**}p < 0.01$, NS, not significant, Student's t test.

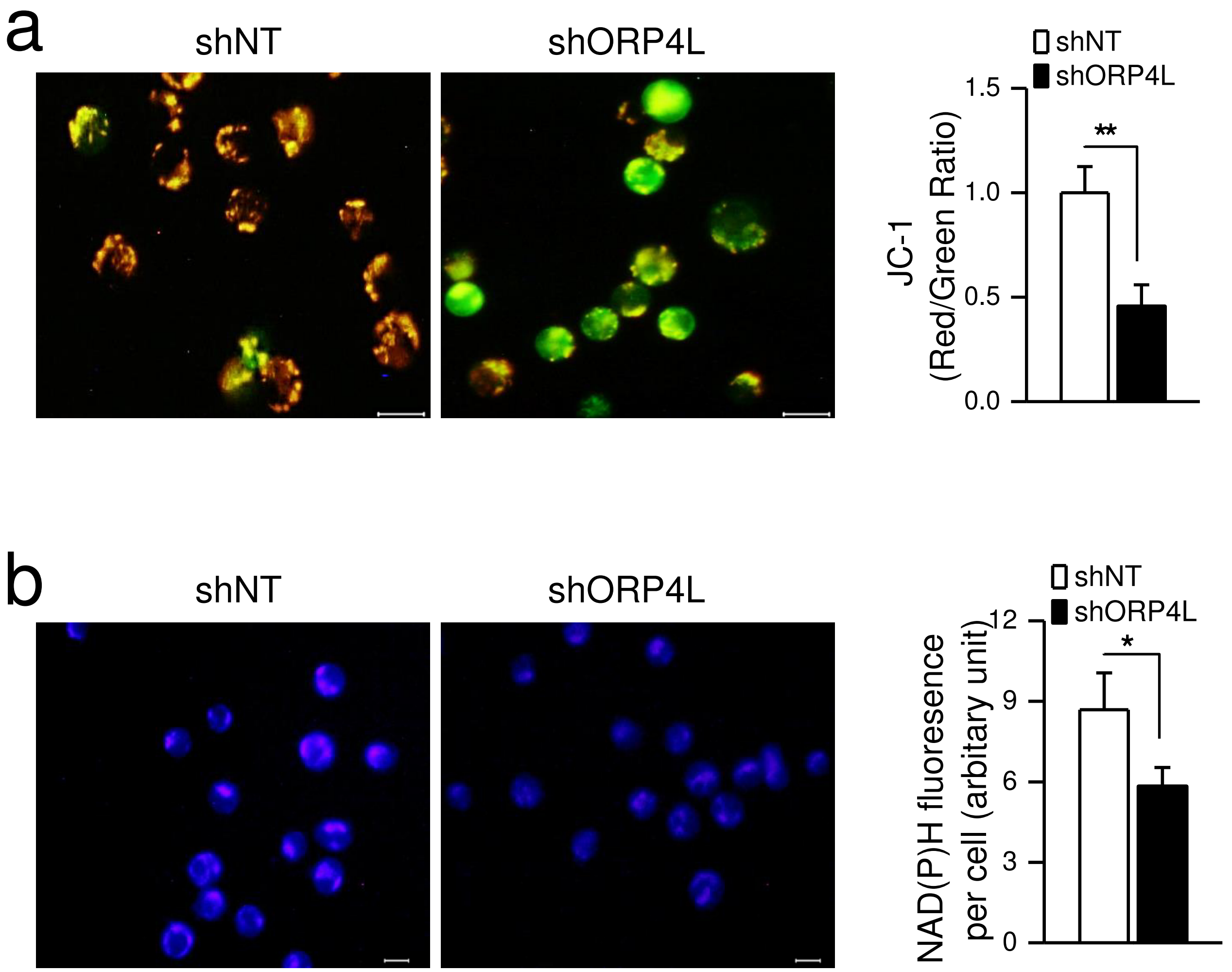

Supplementary Figure 11. ORP4L knockdown reduces the mitochondrial membrane potential and NAD(P)H autofluorescence in Jurkat T-cells. (a) Mitochondrial membrane potential was visualized with epifluorescence microscopy and expressed as ratio of JC-1 aggregates (red) and monomer (green) (Red/Green). **(b)** NAD(P)H autofluorescence was captured with DAPI cube by epifluorescence microscopy and used to calculate FCCP-sensitive mitochondrial NAD(P)H level [ΔNAD(P)H] per cell. Scale bars, 10 μm. The data represent mean ± S.D. (n=3). *$p < 0.05$, **$p < 0.001$, Student's t test.

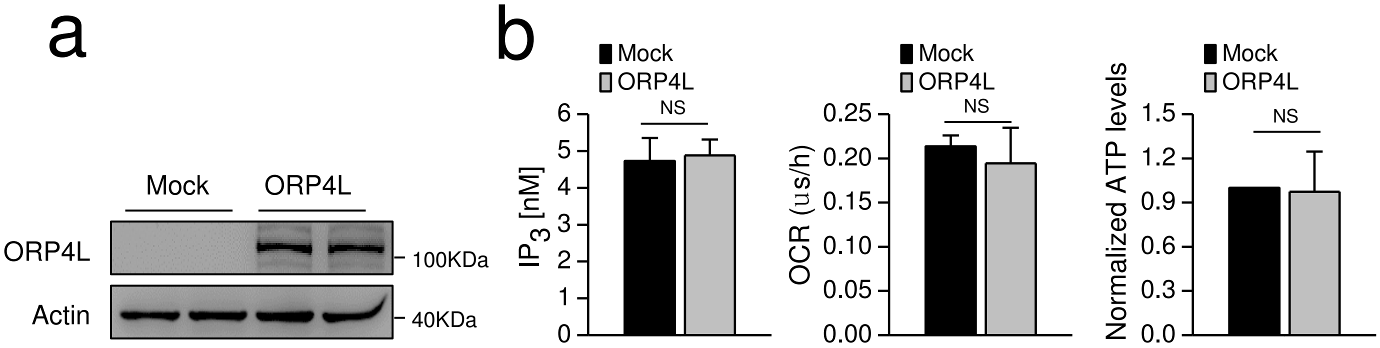

Supplementary Figure 12. ORP4L overexpression failed to affect oxidative phosphorylation in normal T-cells. (a) Western blot verifying the overexpression of ORP4L in transfected T-cells. **(b)** IP_3 production (left), OCR (middle) and ATP levels (right) in control and ORP4L overexpressing T-cells. The data represent mean $\pm$ S.D. value from an experiment performed in triplicate. NS, not significant, Student's t test.

Fig. 1f

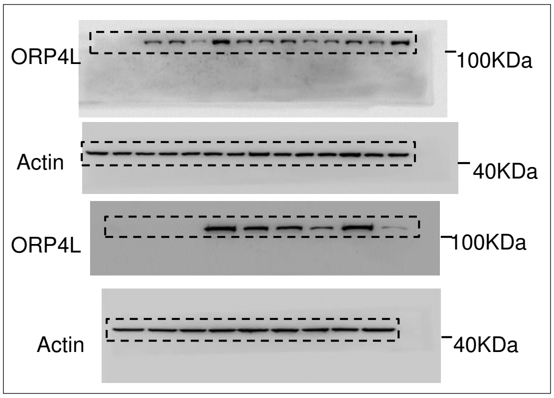

Fig. 1g

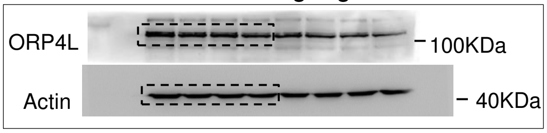

Fig. 2b

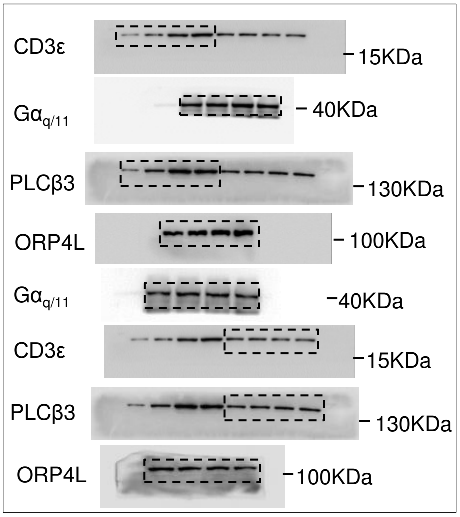

Fig. 2c

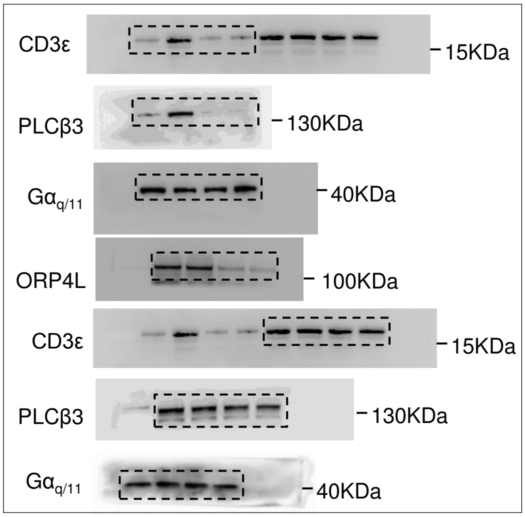

Fig. 2d

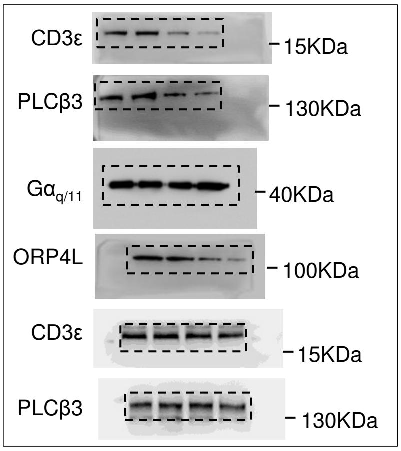

Fig. 2e

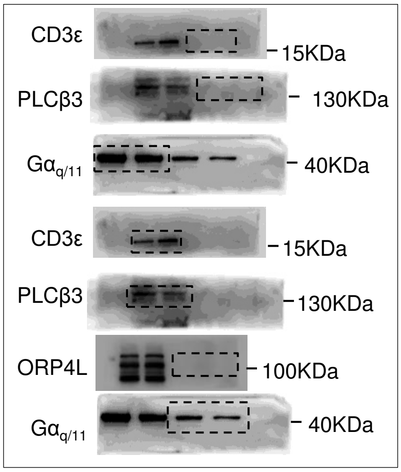

Fig. 3b

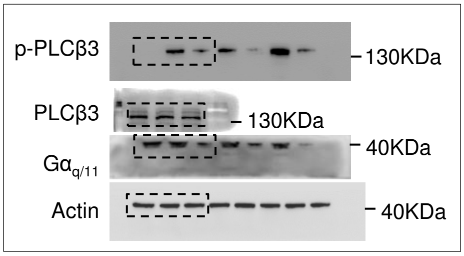

Fig. 3c

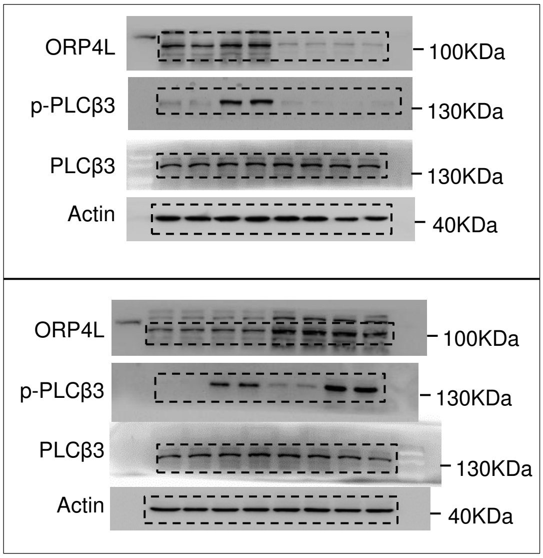

Fig. 3d

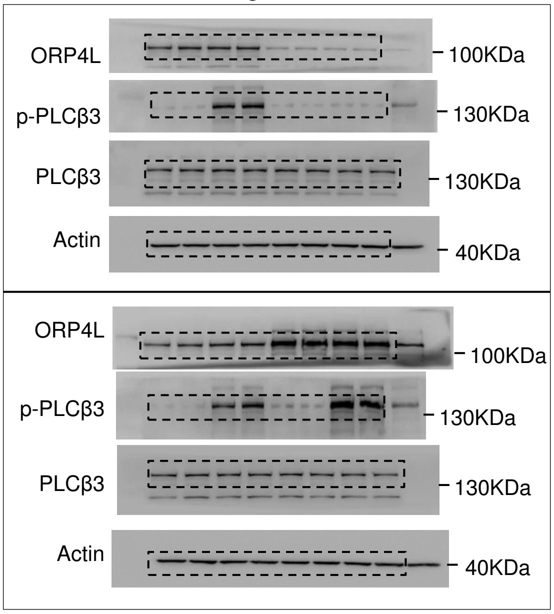

Supplementary Figure 13. Representative original images of immunoblotting results for Figure 1-6. The cropped areas used in the figures are marked by boxes.

Fig. 5a

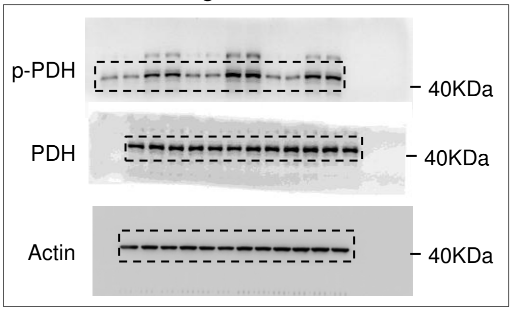

Fig. 5b

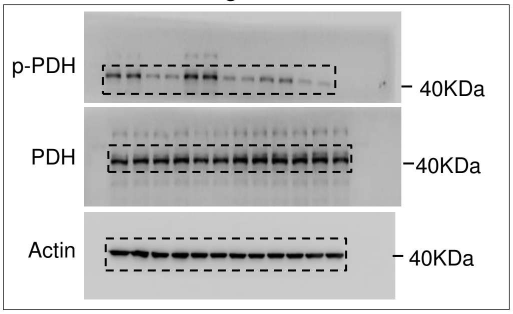

Fig. 5c

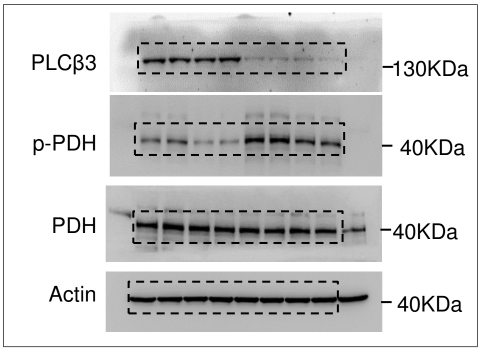

Fig. 5d

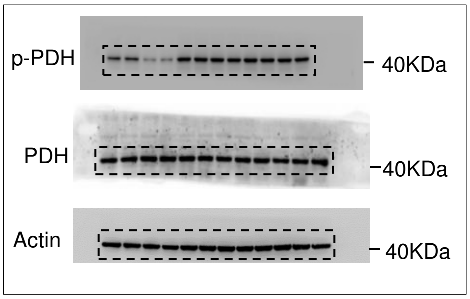

Fig. 5e

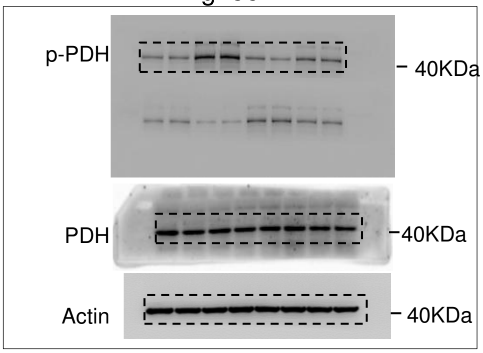

Fig. 5f

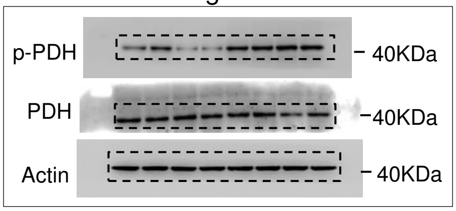

Fig. 5g

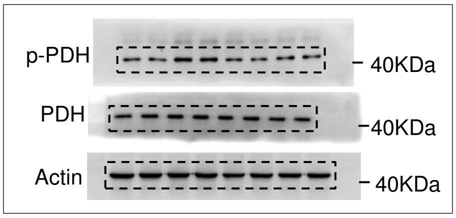

Fig. 5h

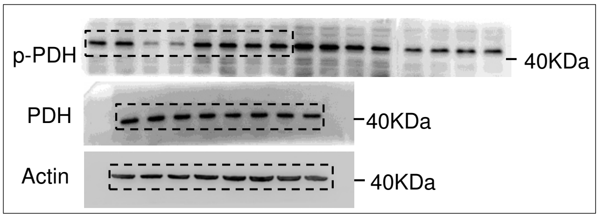

Fig. 6e

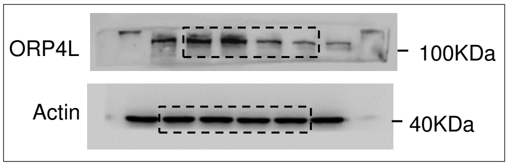

Fig. 6g

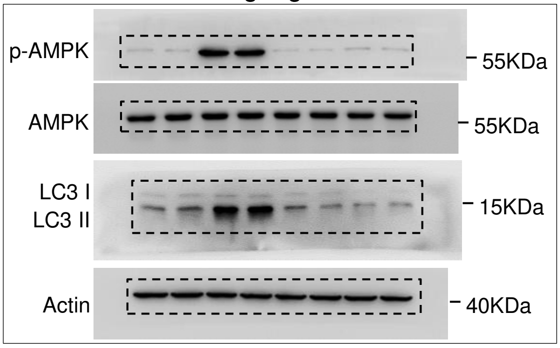

Fig. 6f

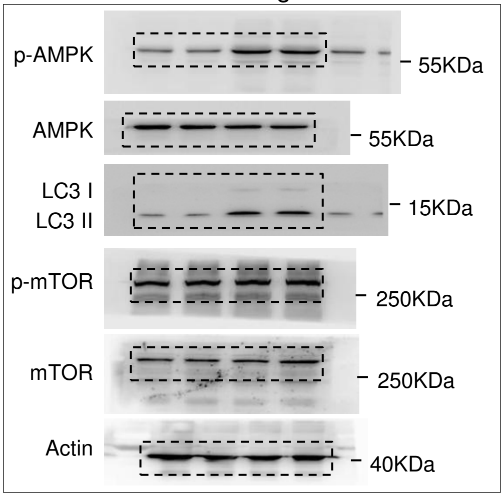

Fig. 6i

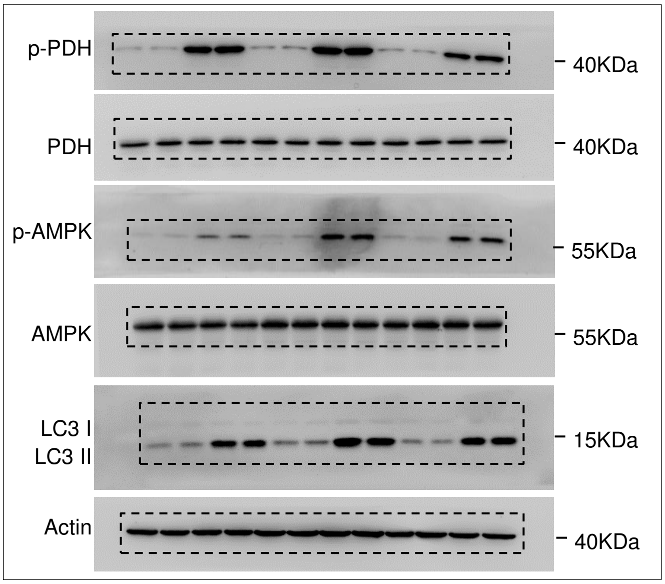

Fig. 6h

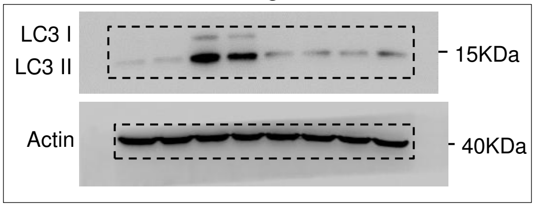

Supplementary Figure 13. Continued

Supplementary Table 1. Summary of the clinical T-ALL cell samples and their analyses

Samples	Age	Sex	Analyses performed											
			ATP	OCR	ECAR	q-PCR	Western blot	IP_3	Ca^{2+}	Cofocal image	Cell death	Engraftment	ROS	PDH/AMPK activity, autophagy
T-ALL 1	36	M	Yes	Yes	Yes	Yes	Yes	Yes	Yes		Yes		Yes	Yes
T-ALL 2	21	F	Yes	Yes	Yes	Yes	Yes	Yes	Yes	Yes	Yes	Yes	Yes	Yes
T-ALL 3	48	M	Yes	Yes	Yes	Yes	Yes	Yes	Yes		Yes		Yes	Yes
T-ALL 4	55	M				Yes	Yes				Yes			
T-ALL 5	20	F	Yes			Yes	Yes				Yes			
T-ALL 6	28	F	Yes	Yes	Yes	Yes	Yes				Yes			
T-ALL 7	61	M				Yes	Yes							
T-ALL 8	37	M	Yes			Yes	Yes				Yes			
T-ALL 9	33	F				Yes	Yes							
T-ALL 10	19	M				Yes	Yes							
T-ALL 11	44	M				Yes	Yes							
T-ALL 12	28	F				Yes	Yes							
T-ALL 13	38	M				Yes	Yes							
T-ALL 14	24	F	Yes	Yes	Yes	Yes	Yes				Yes			
T-ALL 15	39	F				Yes	Yes							
T-ALL 16	18	F				Yes	Yes				Yes			
T-ALL 17	40	M	Yes	Yes	Yes	Yes	Yes				Yes			
T-ALL 18	33	M				Yes	Yes							

Supplementary Table 2. Putative ORP4L-interacting proteins

Symbol	Entrez Gene Name
HCLS1	Hematopoietic lineage cell-specific protein
DARS	Aspartyl-tRNA synthetase
A1S9T	Ubiquitin-like modifier-activating enzyme 1
**CD3E**	**T-cell surface glycoprotein CD3 epsilon chain**
RPL27	60S ribosomal protein L27
JUP	Putative uncharacterized protein ENSP00000382160
CGI-55	PAI1 RNA-binding protein 1
**PLCβ3**	**phosphatidylinositol-4,5-bisphosphate phosphodiesterase beta-3**
FUBP2	Far upstream element-binding protein 2
EVL	Ena/vasodilator-stimulated phosphoprotein-like
RABEP2	Rab GTPase-binding effector protein 2
**Gαq/11**	**Guanine nucleotide binding protein, alpha q/11**
NUDC	Nuclear distribution protein C homolog
DRBF	Double-stranded RNA-binding protein 76

Supplementary Table 3. The targeted shRNA sequences used

Construct	sense 5’- 3’	Anti-sense 5’- 3’
shNT	GCATTGGTCGTCTCT ATTA	TAATAGAGACGACCAATGC
shORP4L	TCAGAGTCAAGCTCAGGTGTA	TACACCTGAGCTTGACTCTGA
shPLCβ3	AGATGAGGGACAAGCATAAGAAGGA	TCCTTCTTATGCTTGTCCCTCATCT
shPLCγ1	AAACAACCGGCTCTT CGT	ACGAAGAGCCGGTTGTTT
siGq/11	GGAGUACAAUCGGUCUAAUU	UUAGACCAGAUUGUACUCCUU

Supplementary Table 4. Oligonucleotide primers used for cDNA constructs

Construct	Forward primer 5'- 3'	Reverse primer 5'- 3'
ORP4L-pcDNA4 HisMaxC	ATTtctagaATGGGGAAAGCGGCGGT*	ATTtctagaGTGGCGCTCAGAAGATGTTG GGGCACATATGCCA
ORP4M-pcDNA4 HisMaxC	ATTagatctATGTCGTCACTGGCAGCGAAG	ATTgtcgacGAAGATGTTGGGGCACATATG
ORP4S-pcDNA4 HisMaxC	ATTagatctATGGAAGACTCCACATCCTTCA	ATTgtcgacGAAGATGTTGGGGCACATATG
ORP4L(ΔPH)-pcDNA4HisMaxC	**Forward 1:** ATTagatctATGGGGAAAGCGGCGGCT **Forward 184:** GCTGTCCAGAGGCAGTAAGGCGTCCCCAGA GTCATCTGAA	**Reverse 916:** ATTgtcgacGAAGATGTTGGGGCACATATGCCA **Reverse 281:** TTCAGATGACTCTGGGGACGCCTTACTGCCTCT GGACAGC
ORP4L(ΔFFAT)-pcDNA4HisMaxC	**Forward 1:** ATTagatctATGGGGAAAGCGGCGGCT **Forward 450:** TGATGAAGGATGTGGAGTCTTCCATGGTATC TTCATCTTCCTCACTGTCC	**Reverse 916:** ATTgtcgacGAAGATGTTGGGGCACATATGCCA **Reverse 465:** GGACAGTGAGGAAGATGAAGATACATGGAAGA CTCCACATCCTTCATCA
ORP4L(PH)-pcDNA4HisMaxC	ATTagatctGTGAGGGCTGGCTTCTCAAGT	ATTgtcgacCTTGGCCAGCTCCAGGGCGGTGAT
PLCβ3-pcDNA4HisMaxC	ATTgaattcATGGCGGGCGCCCAGCCCGGC	ATTctcgagTCAGAGCTGCGTGTTCTCCT
PLCγ1-pcDNA4HisMaxC	ATTgaattcATGGCGGGCGCCGCGTCCCCTT	ATTtctagaCTAGAGGCGGTTGTCTCCATTGACC

*Restriction sites are indicated in lower case letters.
